# An integrated transcriptome and metabolome analysis reveals the gene network regulating flower development in *Pogostemon cablin*

**DOI:** 10.3389/fpls.2023.1201486

**Published:** 2023-06-29

**Authors:** Chan Zhang, Xiaofeng Liu, Ya Liu, Jing Yu, Guanglong Yao, Huageng Yang, Dongmei Yang, Yougen Wu

**Affiliations:** ^1^ Sanya Nanfan Research Institute of Hainan University, College of Tropical Crops, Hainan University, Sanya, China; ^2^ Guangdong VTR BioTech Co., Ltd., Zhuhai, China

**Keywords:** *Pogostemon cablin*, flower development, GA and auxin signaling, MIKC-MADS, coexpression network, correlation analysis

## Abstract

*Pogostemon cablin* is a well-known protected species widely used in medicine and spices, however the underlying molecular mechanisms and metabolite dynamics of *P. cablin* flower development remain unclear due to the difficulty in achieving flowering in this species. A comparison of the transcriptome and widely targeted metabolome during *P. cablin* flower development was first performed in this study. Results showed that a total of 13,469 differentially expressed unigenes (DEGs) and 371 differentially accumulated metabolites (DAMs) were identified. Transcriptomic analysis revealed that the DEGs were associated with starch and sucrose metabolism, terpenoid biosynthesis and phenylpropanoid biosynthesis. Among these DEGs, 75 MIKC-MADS unigenes were associated with the development of floral organs. Gibberellins (GAs), auxin, and aging signaling might form a cross-regulatory network to regulate flower development in *P. cablin*. According to the metabolic profile, the predominant DAMs were amino acids, flavonoids, terpenes, phenols, and their derivatives. The accumulation patterns of these predominant DAMs were closely associated with the flower developmental stage. The integration analysis of DEGs and DAMs indicated that phenylpropanoids, flavonoids, and amino acids might be accumulated due to the activation of starch and sucrose metabolism. Our results provide some important insights for elucidating the reproductive process, floral organ, and color formation of *P. cablin* flowers at the molecular level. These results will improve our understanding of the molecular and genetic mechanisms involved in the floral development of *P. cablin*.

## 1 Introduction

*Pogostemon cablin* Benth, a species of the Lamiaceae family, is a crucial aromatic plant famous for its volatile oils (Li et al., 2011; Swamy and Sinniah, 2016). It’s mainly cultivated in tropical and subtropical regions, such as Malaysia, China, and Philippines (Swamy and Sinniah, 2015). As a traditional Chinese medicine, *P. cablin* was listed in the first batch of “Lingnan Traditional Chinese Medicine Protected Species” in Guangdong Province, China, in 2017. The main active components of essential oil are sesquiterpene, including patchoulol, which decide the quality of *P. cablin* (Shen et al., 2022; Yan et al., 2022). In the biosynthesis of patchoulol, the isopentenyl diphosphate (IPP) and dimethylallyl diphosphate (DMAPP) are biosynthesized by the mevalonate (MVA), followed by the condensation to form farnesyl diphosphate (FPP). Finally, the FPP is conversed to patchoulol with the terpene synthases (TPSs), which is responsible for the production of patchouli oil (Yu et al., 2015; Shen et al., 2022). Besides leaves, the inflorescences of *P. cablin* also biosynthesize and accumulate large amounts of patchoulol, which are composed of 17 sesquiterpenes (97.7%) (Verma et al., 2019). However, a phenomenon of rare flowering accompanied by a lack of seeds has been observed in *P. cablin* in many countries, such as China, India and Cuba (Li et al., 2011; Swamy and Sinniah, 2016). Due to this phenomenon, the large-scale propagation and preservation of *P. cablin* on farms occurs mainly by cottage propagation (Swamy and Sinniah, 2016), resulting in the accumulation of viruses and root-knot nematodes, and decreases in the resistance and quality of *P. cablin.* This phenomenon seriously impedes the cultivation and development of *P. cablin*. Hence, an understanding of the flowering process of *P. cablin* is urgently needed to promote the breeding of this species and the production of patchouli oil.

Flowering involves the following two main steps: first, the shoot apex meristem changes from a vegetative state to a reproductive state driven by the perception and integration of floral induction signals; and second, the shoot apex differentiates into an inflorescence or flower (Satish and Manju, 2018). Floral induction is coordinated by six pathways, including the photoperiod, gibberellin (GA), autonomy, aging, temperature, and vernalization (Wellmer and Riechmann, 2010). In recent years, approximately 180 genes were identified to play an important role in flower development in *Arabidopsis thaliana* (Fornara et al., 2010; Ó’Maoiléidigh et al., 2014). According to the flowering process, these genes were divided into three main clades (Satish and Manju, 2018). The first clade comprises flower pathway integrators, including FLOWERING LOUCS T (*FT*) and SUPPRESSOR OF OVEREXPRESSION OF CONSTANS (*SOC1*); the second clade comprises floral meristem identity genes, such as APETALA1 (*AP1*) and FRUITFUL (*FUL*); and the third clade comprises floral organ identity genes, such as, APETALA2 (*AP2*), SEPALLATA (*SEP*) and AGAMOUS (*AG*) (Satish and Manju, 2018). For most flowering plants, ABCDE model play a key role in the flower development process. In this model, different organ developments are regulated by various genes, such as sepals (A + E), petals (A + B + E), stamens (B + C + E), carpels (C + E), and ovules (D + E). In addition, class A contains APETALA1 (AP1) and FRUITFULL (FUL); class B contains PISTILLATA (PI) and APETALA3 (AP3); class C contains AGAMOUS (AG); class D contains SEEDSTICK (STK) and class E contains SEPALLATA genes (SEP1, SEP2, SEP3, and SEP4). Interesting, most of these genes are belonging to MADS-box gene family (Satish and Manju, 2018; Ren et al., 2021). These genes work together to complete the transition of the shoot apex meristem into a flower. Then, it enters the bud stage and the full-bloom stage successively, followed by the fruit- or seed-formation stage.

*P. cablin* is also a flowering plant that naturally blooms after the beginning of spring, and the flowering period lasts around 30-42 days (Verma et al., 2019). *P. cablin* flowers are indeterminate inflorescences whose terminal inflorescence maintains the state of the meristem. Although the genomes of *P. cablin* have been published (He et al., 2018; Shen et al., 2022), limited studies have investigated *P. cablin* flowers due to their morphological structure (Li et al., 2011) and essential oil composition (Verma et al., 2019). However, the available information on the molecular mechanisms and metabolite dynamics of *P. cablin* flower development is scarce due to the flowering difficulties of this species. Herein, the transcriptomic and metabolic profiles during flower development in *P. cablin* were characterized using transcriptomic and widely targeted metabolomic technologies. This study had the following two objectives: (1) to verify the regulation pathways and genes of flower development and (2) to determine the dynamics of metabolites during flower development in *P. cablin*. This report provides the first description of the molecular genetic mechanisms of *P. cablin* flower development and provides a basis for further studies on *P. cablin* sexual reproduction and breeding.

## 2 Materials and methods

### 2.1 Materials and pretreatment

*P. cablin* (Nan Xiang, grown in Hainan) was cultivated at the germplasm resource garden of Hainan University (20°05′78′′N, 110°31′90′′E). Twelve mixed samples were collected from 7-month-old plants at four different flower stages on April 3, 2020, including the inflorescence-bearing meristem stage (F1), flower bud stage (F2), full-bloom stage (F3) and withered flower stage (F4). Three replicates were collected at each flowering stage. The harvested samples were frozen immediately in liquid nitrogen. Half of the samples were stored at -80 °C for RNA extraction, and the others were freeze-dried for chemical component identification.

### 2.2 RNA extraction, cDNA library construction and sequencing

Total RNA was extracted using TRIzol^®^-Reagent according to the manufacturer’s instructions. After quality check, the high-quality RNA samples (OD260/280 = 1.8~2.2, RIN ≥ 8.0) were used to construct the RNA-seq library, according to the TruSeq™ instructions. First, the polyA selection method was performed to isolate mRNA, which was then segmented using lysis buffer. Second, cDNAs were obtained using a SuperScript Double-Stranded cDNA Synthesis Kit. Illumina adapters were then ligated to the cDNAs, and amplification fragments (200 bp) were obtained by PCR. After quantification with TBS380, the paired-end cDNA libraries were sequenced with a NovaSeq 6000 sequencer (Illumina) (2 × 150 bp read length). The raw data were submitted to the NCBI Short Read Archive database under accession number PRJNA769458.

### 2.3 Data filtering, transcript assembly and gene functional annotation

Reads with a low quality (Q < 20) or containing more than 10% N bases were excluded using Trimmomatic-0.39.jar (Yan et al., 2022). The *P. cablin* genome sequence, including the coding DNA sequence (CDS), protein sequences and GFF3 files, was downloaded from the figshare database (https://figshare.com/). High-quality reads were mapped to the *P. cablin* genome using HISAT2 software (He et al., 2018). The NCBI RefSeq, Swiss-Prot, Gene Ontology (GO), Kyoto Encyclopedia of Genes and Genomes (KEGG) and Pfam databases, were used for functional annotation *via* DIAMOND V0.9.24 (Buchfink et al., 2021).

### 2.4 Verification and functional enrichment analysis of differentially expressed unigenes

The transcript levels of the unigenes were quantified based on the TPM reads system to identify the DEGs between the two flower periods. The transcript abundance was determined using RSEM (Li et al., 2011). DEGs (log2FC > 2 or < -2 and p value < 0.001) were screened using DESeq2 version 2.10 (Yan et al., 2022). Correlations were determined by calculating Pearson’s correlation coefficient (r). GO and KEGG enrichment analyses (p < 0.05 and FDR < 0.05) were performed using GOATOOLS and KOBAS software (version 2.0), respectively (Xie et al., 2011).

### 2.5 Identification and phylogenetic analysis of MADS genes

The sequences of the SRF (Pfam: PF00319) and K-box domains (Pfam: PF01486) served as queries to search the *P. cablin* genome and affirm MADS genes using HMMER (version 3.3.2). Phylogenetic relationships among MADS genes were aligned using Clustal Omega (https://www.ebi.ac.uk) and IQ-tree (http://iqtree.cibiv.univie.ac.at/) with the method of Maximum likelihood (ML), followed by visualizion with iTOL (https://itol.embl.de/).

### 2.6 Extraction and detection of metabolites using LC-MS

Ten milligrams of sample were used to extract metabolites with the extract solution (methanol: water = 3:1) and then filtered. Metabolites in 2 μL were separated using an ExionLC system (AB SCIEX) equipped with a Waters UPLC HSS T3 column. The temperatures of the column and autosampler were 40 °C and 4 °C, respectively. In the analysis procedure, the gradient program (A, 0.1% formic acid: B, acetonitrile) was as follows: 98:2 (V/V) at 0-0.5 min, 50:50 (V/V) at 10.0 min, 5:95 (V/V) at 11.0-13.0 min, and 98:2 (V/V) at 13.1-15 min. The flow rate was 400 μL·min^-1^, and a SCIEX Q Trap 6500+ instrument (AB SCIEX Technologies) was used for the analysis.

### 2.7 Bioinformatics analysis of metabolomic data

SCIEX Analyst Work Station Software (1.6.3) was employed to clean the MRM data. Metabolite peak detection and annotation were performed using the R program. Principal component analysis (PCA), orthogonal partial least squares differential analysis (OPLS-DA) and hierarchical clustering analysis were performed using SIMCA Software (V16.0.2). KEGG enrichment analysis was conducted using the OmicShare platform (https://www.omicshare.com/), and pathways with p < 0.05 and FDR < 0.05 were identified as significantly different metabolic pathways. The heatmap was visualized using Morpheus (https://software.broadinstitute.org/).

### 2.8 Validation of transcriptomic data

To test the validation of transcriptomic data, 18 DEGs were chose and quantified at four flowering stages. qRT-PCR was performed using SYBR Green qPCR Mix in the Applied Biosystems device (Thermo Fisher). The relative transcript levels were computed with the 2^−ΔΔCT^ method. All the primers used for qRT-PCR analysis are mentioned in **Table S11**.

### 2.9 Coexpression network construction

A weighted gene correlation network analysis (WGCNA) was employed using a freely accessible R package (version 1.70-3) with the default parameters according to the protocol designed to identify coexpressed genes (Langfelder and Horvath, 2008). TOM similarity was also calculated, and correlation variables with TOM > 0.1 were considered correlated. A graphical representation of the coexpression network was constructed using Cytoscape v 3.8.1 (Mauceri et al., 2021).

### 2.10 Transcriptome and metabolome integrated correlation analysis

A transcriptome and metabolome integrated correlation analysis was conducted by MetaboAnalyst 5.0 (Pang et al., 2021). Spearman’s rank correlation analysis was performed using the R package based on the DEGs and DAMs. Genes and metabolite network plots were constructed using Cytoscape v3.8.1. The correlation coefficients of DEGs and DAMs (value> 0.80 or < -0.80, and p < 0.05) was displayed in the network plot for clarity.

## 3 Results

### 3.1 Dynamic transcriptomic profiles during flower development in *P. cablin*

**Figure 1:**
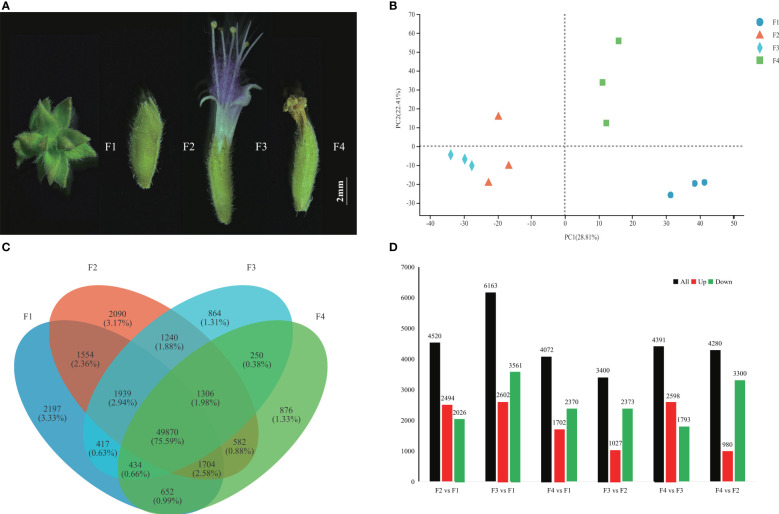
Transcriptome profiles of *P. cablin* flowers at four stages. **(A)** Four flower stages of *P. cablin* (from left to right): the inflorescence-bearing meristem stage (F1), flower bud stage (F2), full-bloom stage (F3), and withered flower stage (F4). The white line represents 2 mm. **(B)** PCA plot showing the gene expression patterns at the four flower stages. PC1 and PC2 represent the first principal component and the second principal component, respectively. **(C)** Venn diagram showing the expression profile of flower stage-specific unigenes. **(D)** Numbers of DEGs in different comparison sets, including F2 vs. F1, F3 vs. F1, F4 vs. F1, F3 vs. F2, F4 vs. F2, and F4 vs. F3. The black color indicates DEGs, the red color indicates upregulated unigenes, and the green color indicates downregulated unigenes.

To identify the candidate genes connected with *P. cablin* flower development, 12 libraries were generated at the inflorescence-bearing meristem stage (F1), flower bud stage (F2), full-bloom stage (F3), and withered flower stage (F4) (**Figure 1A**). The transcriptomic data in each library were 7.67 G on average, with low average error rate (0.024%) and high Q30 (94.72%), indicating reliable databases (**Table S1**). A total of 109,498 unigenes were obtained from these databases. PCA displayed a similar gene expression pattern in the same flower stage, which were divided by PC1 (28.81%) and PC2 (22.41%) (**Figure 1B**). Venn diagrams showed that the overlap between F1 and F2 consisted of 1,554 (2.36%) individual unigenes, while only 250 (0.38%) unigenes were overlapped between F3 and F4 (**Figure 1C**). Approximately 75.59% of unigenes were shared during four stages. More stage-specific genes were identified in the F1 (2,197 unigenes) and F2 (2,090 unigenes) than in the F3 (864 unigenes) and F4 (876 unigenes) libraries (**Figure 1C**). A total of 13,469 DEGs were confirmed by DESeq2 (log_2_FC>2, P < 0.001) (**Table S2**). Notably, 4,520, 6,163, and 4,072 DEGs were identified in the F2 vs. F1, F3 vs. F1 and F4 vs. F1 comparison sets, respectively (**Figure 1D**).

### 3.2 Functions of DEGs involved in *P. cablin* flower development

**Figure 2:**
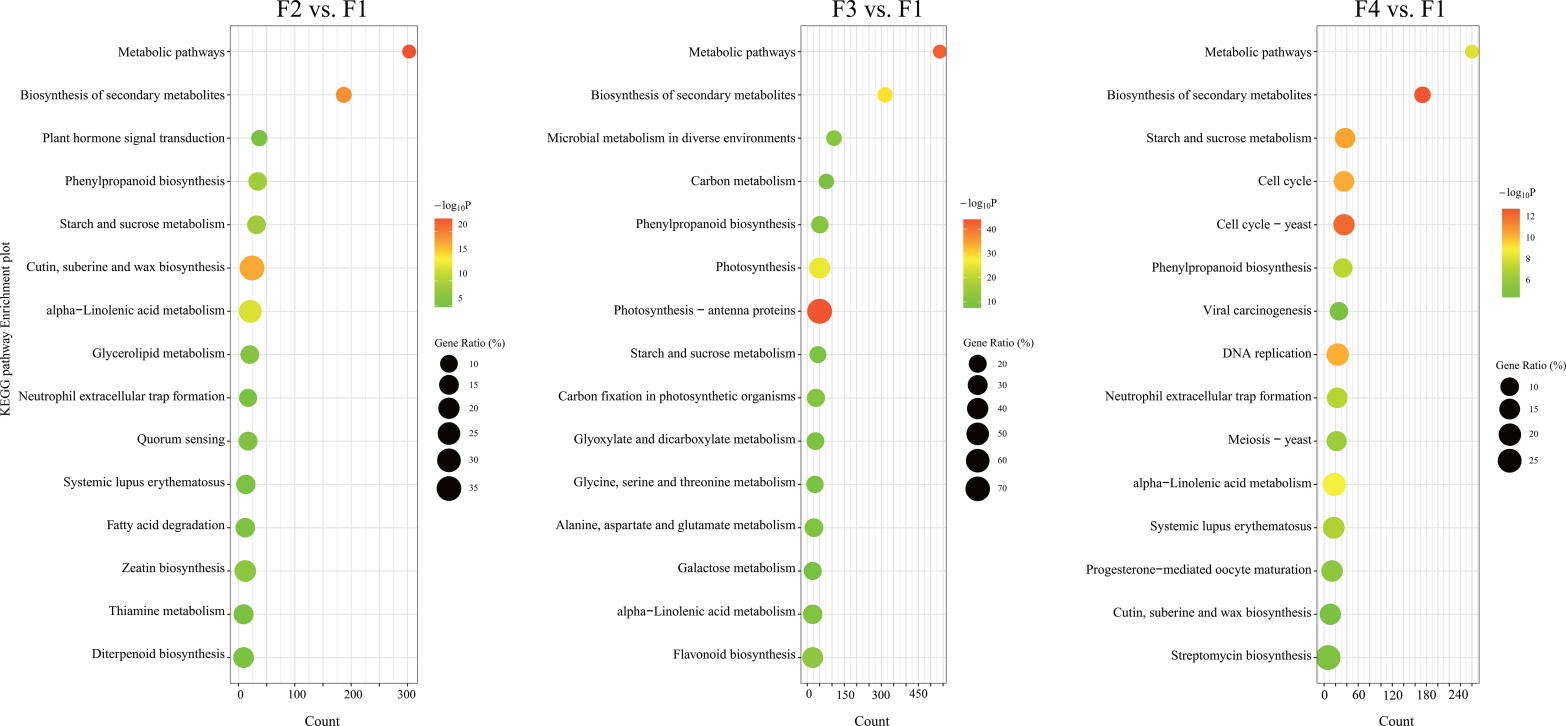
KEGG pathway enrichment analysis of DEGs at different flowering stages of *P. cablin*. KEGG pathway enrichment analysis of DEGs in three comparison sets: the flower bud stage (F2) vs. the inflorescence-bearing meristem stage (F1), the full-bloom stage (F3) vs. the inflorescence-bearing meristem stage (F1), and the withered flower stage (F4) vs. the inflorescence-bearing meristem stage (F1). The top fifteen significantly different metabolic pathways (p < 0.05, FDR < 0.05) are shown in the diagram. The percentage indicates the number of DEGs out of the total genes in the corresponding KEGG pathway. The size of the bubble represents the number of DEGs in the pathway; the color range indicates the significance of pathway enrichment.

GO and KEGG enrichment analyses showed the top 15 significantly different metabolic pathways (p < 0.05 and FDR < 0.05) in the **Figures 2**, **S1**. According to the GO functional analysis, 3,590, 4,927, and 3,227 DEGs from the three comparison sets were annotated to 4,139, 4,840, and 4,066 GO terms and were significantly enriched in 457, 667, and 510 GO terms, respectively (**Table S3**). Among them, the terpenoid biosynthesis process and anthocyanin-containing compound biosynthesis process were significantly more active from F2 to F4 (**Figure S1**). Moreover, the auxin-activated signaling pathway, pollen exine formation, and sporopollenin biosynthesis process were enriched at F2 (**Figure S1**). Two processes of carbohydrate metabolism, and four important processes involved in cell division and DNA replication, were enriched at F3 and F4, respectively (**Figure S1**). These results indicated that the development of *P. cablin* flowers is accompanied by the biosynthesis of terpenoids and anthocyanins.

The KEGG pathway enrichment analysis showed 1,786, 3,532, and 1,904 DEGs were annotated to 252, 297, and 254 KEGG pathways in the three comparison sets, respectively. Among them, 62, 85, and 86 KEGG pathways were significantly enriched in the F2 vs. F1, F3 vs. F1 and F4 vs. F1 comparison sets, respectively (**Table S4**). The phenylpropanoid biosynthesis, alpha-linolenic acid metabolism, and starch and sucrose metabolism pathways were all significantly enriched from F2 to F4, and the percentage of genes in these pathways was highest at F3 (**Figure 2**; **Table S4**). Furthermore, flavonoid biosynthesis and the plant hormone signal transduction pathway were also significantly enriched at F2 and F3 (**Figure 2**; **Table S4**), and the diterpenoid biosynthesis pathway was enriched at F2. DEGs involved in four pathways associated with carbohydrate metabolism were significantly enriched at F3. Similarly, four pathways associated with cell proliferation, including the cell cycle, meiosis and DNA replication were active at F4. All of pathways associated with growth, pigmentation and energy costs, displayed some distinctions in the stages of *P. cablin* flower development.

Hormone signaling pathways are important for flower development. The GO and KEGG enrichment results showed that auxin signal transduction was clearly enriched during *P. cablin* flower development (**Figure S1**; **Table S4**). Twenty-nine DEGs were related to the auxin signaling pathway (**Figure S2**). For instance, most genes including *AUX/LAX*, *IAA*, *ARF*, and *SAUR*, were upregulated from the F2 to F3 stage, while the transcript levels of *GH3* genes decreased at the F2 and F3 stages (**Figure S2**). Thus, the activation of auxin signaling at the bud flower and full-bloom stages might favor *P. cablin* flower development.

### 3.3 Identification of differentially expressed terpene biosynthesis-related genes

**Figure 3:**
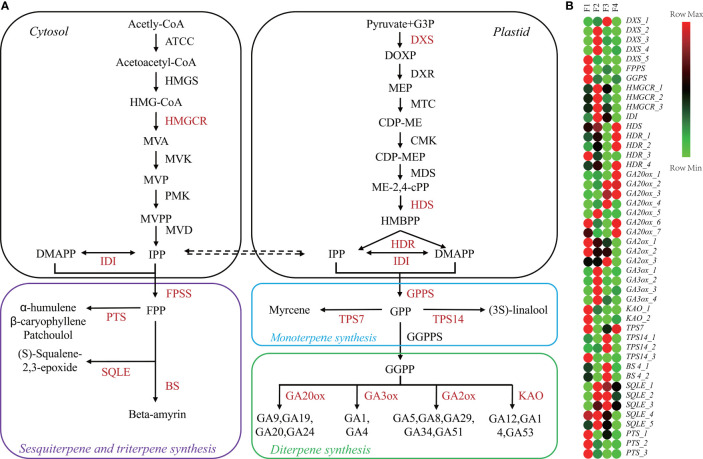
Changes in terpene metabolism-related genes during *P. cablin* flower development. **(A)** Diagram of the terpene biosynthesis pathway. **(B)** Heatmap showing the expression levels [log_2_(TPM+1)] of genes related to terpenoid biosynthesis during flower development. The pathway was redrawn based on ko0900, ko00902, ko00904, and ko00909 in the KEGG database (https://www.kegg.jp/). F1, inflorescence-bearing meristem stage; F2, flower bud stage; F3, full-bloom flower stage;and F4, withered flower stage.

As main active ingredients, terpenes are synthesized through methyl-erythritol phosphate and the mevalonate pathways (**Figure 3A**). KEGG analysis showed that 210 terpenoid synthesis-related genes were identified (**Table S5**), including 139 genes in the terpenoid backbone, 8 genes in monoterpenoids, 43 genes in diterpenoid biosynthesis, and 20 genes in sesquiterpenoid and triterpenoid biosynthesis (**Table S5**). We then identified 59 DEGs encoding 21 enzymes associated with terpenoid biosynthesis (**Table S6**). The p values and FDR values of these DEGs in the F2 vs. F1, F3 vs. F1 and F4 vs. F1 comparison sets are listed in **Table S7**.

A heatmap of 46 DEGs associated with terpenoid biosynthesis during the four flower stages was generated (**Figure 3B**). According to the heatmap, *FPPS*, *GGPPS*, *KAO_1-2*, *GA2ox_1-2* and *TPS7* were highly expressed at F1, while the expression levels of *TPS14_1-2* and *BS4_1-2* were increased at F3 (**Figure 3B**). The highest levels of *HMGCR_1-3*, *IDI* and *GA3ox_1-4* transcripts were detected at F2. Patchoulol, a major component of *P. cablin*, is catalyzed by the enzyme patchoulol synthase (PTS). The *PTS_1-3* genes were highly expressed at F1. Interestingly, 16 key GA-related genes such as *KAO*, *GA2ox*, *GA3ox*, and *GA20ox*, were differentially expressed throughout the process of flower development. Taken together, these results suggested that GA signaling might be involved in *P. cablin* flower development.

### 3.4 Identification of DEGs associated with flower development

Two hundred thirty-seven DEGs related to flower development were characterized (**Table S10**). These DEGs were sorted into six clades based on flowering pathway, including 55 DEGs in the photoperiod/circadian pathway, 50 DEGs in the GA signaling, 29 DEGs in the auxin signaling, and 13 DEGs in the aging pathway. Nine and five DEGs were identified in the nutrient pathway and the vernalization pathway, respectively. Seventy-six DEGs were associated with flower pathway integration-related and floral organ identity genes, such as *FT*, *SOC1*, *AP1*, *AG*, PISTILLATA (*PI*) and *SEP* (**Table S10**).

**Figure 4:**
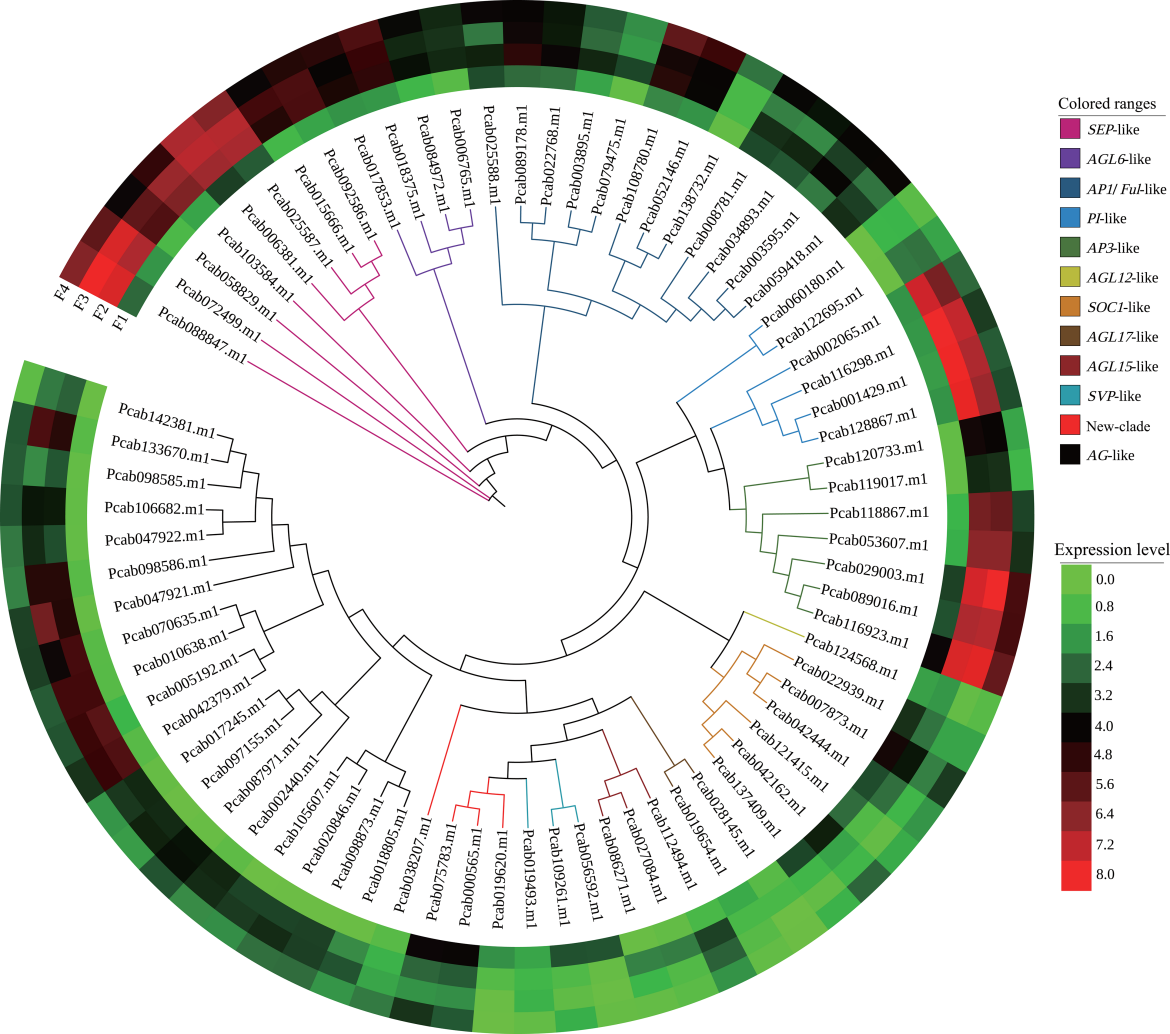
Phylogenetic and expression analyses of MIKC-MADS genes from *P. cablin*. Classification of differentially expressed MIKC-MADS genes identified in *P. cablin*. The phylogenetic tree was constructed using IQ-tree and EvolView. Heatmap showing the expression levels [log_2_(TPM+1)] of MIKC-MADS genes during flower development at the inflorescence-bearing meristem stage (F1), flower bud stage (F2), full-bloom stage (F3), and withered flower stage (F4).

Overall, 270 MADS genes, including 132 MIKC-type and 138 type I MADS genes, were identified (**Figure S3**). A phylogenetic analysis showed that MIKC-MADS genes were sorted into 12 classes such as *SVP-like*, *AG-like*, and *AP1/FUL-like*. Among them, 80 differentially expressed MADS unigenes (75 MIKC-type, 2 Mα and 3 Mδ MADS) were identified (**Figure S3**; **Table S7**). As expected, 69% of MIKC-MADS genes were involved in the ABCDE model: 12 genes in A-class (*AP1-like*), 13 genes in B-class (*PI-like* and *AP3-like*), 19 genes in C/D class (*AG-like*), and 8 genes in E-class (*SEP-like*) (**Figure 4**). Most genes of the *AG-like*, *AP3-like*, *SEP-like*, *PI-like*, and *AP1-like* clade were upregulated from F2 to F4 compared to F1. However, *SVP-like* and *SOC1-like* clade genes displayed downregulated expression from F2 to F4. Besides MIKC-MADS TF, 13 aging-related differentially expressed *SPL* genes were identified, and *SPL1* and *SPL8* exhibited upregulated expression at F2 and F3, respectively. Moreover, the expression levels of *SPL3*, *SPL7*, *SPL13A*, *SPL15*, and *SPL12* were significantly upregulated at the inflorescence-bearing meristem stage (**Figure S4**). These results implied that MIKC-MADS and *SPL* genes might participate in the various phases of *P. cablin* floral organ formation.

### 3.5 Validation of RNA-seq data

**Figure 5:**
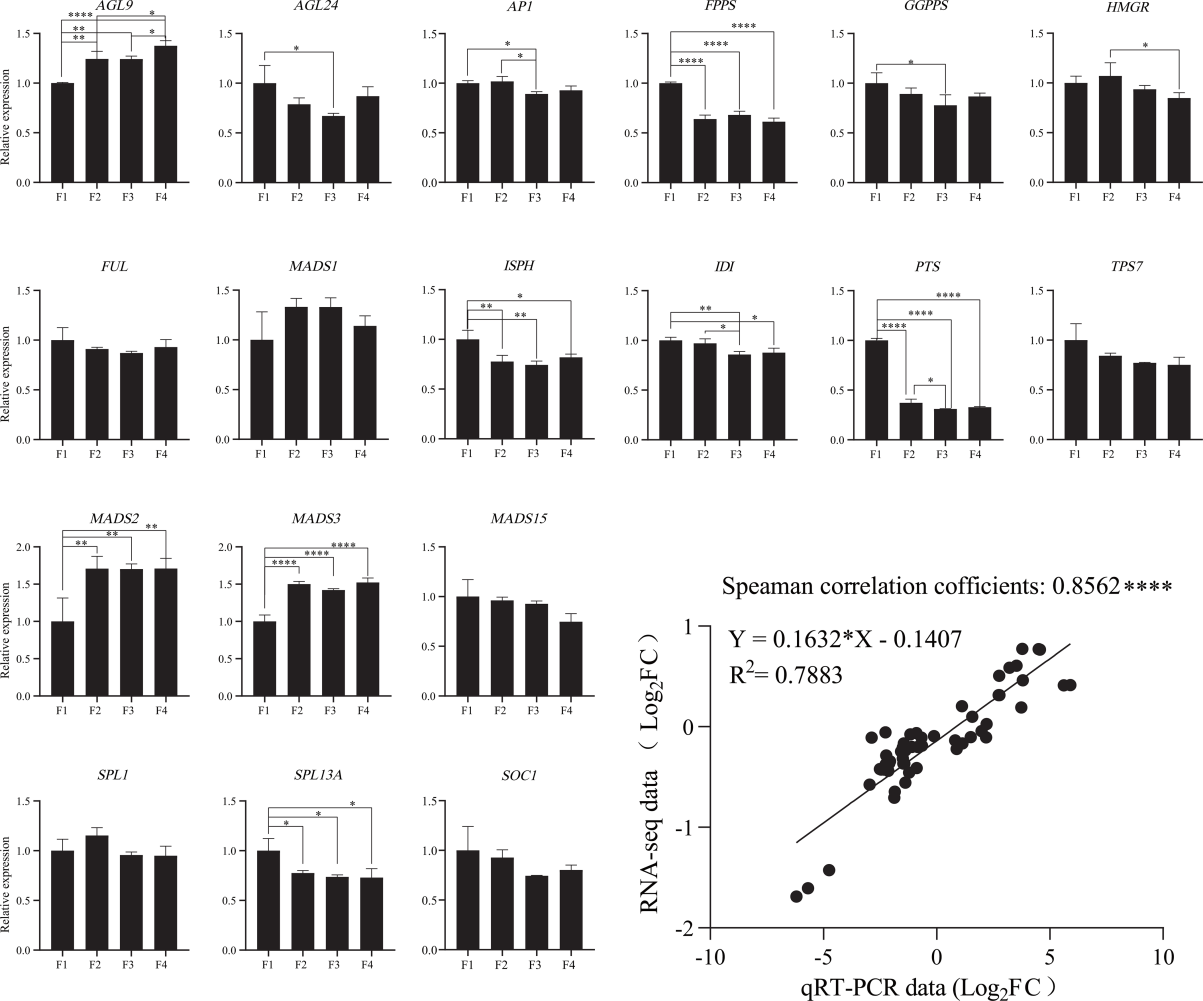
Validation of the expression of terpenoid biosynthesis and flowering-related genes in *P. cablin*. The expression levels of 18 flower development-related genes in four flower stages were validated using qRT-PCR. The relative expression levels of 18 DEGs were calculated using the 2^−ΔΔCt^ method; 18S rRNA served as an internal reference gene. All these data were based on the analysis of three independent biological replicates. Linear regression analysis of the qRT-PCR data and RNA-seq data. “*” indicates significant differences, as determined by one-way ANOVA followed by Tukey’s test (P<0.05). *, P < 0.05; **, P < 0.01; ***, P < 0.001.

To verify the validation of the transcriptomic data, the expression of 18 representative DEGs in the four flower stages were tested using qRT-PCR (**Figure 5**; **Table S11**). These DEGs were related to terpenoid biosynthesis and flower development. The relative expression levels of qRT-PCR and RNA-seq between the two flowering stages were logarithmically processed. Spearman correlation analysis was performed using the above logarithmic results. The Spearman correlation coefficient was 0.8562, indicating that the expression pattern of most transcripts revealed using qRT-PCR was consistent with the RNA-seq data (**Figure 5**).

### 3.6 Metabolic profiles at different flower development stages

**Figure 6:**
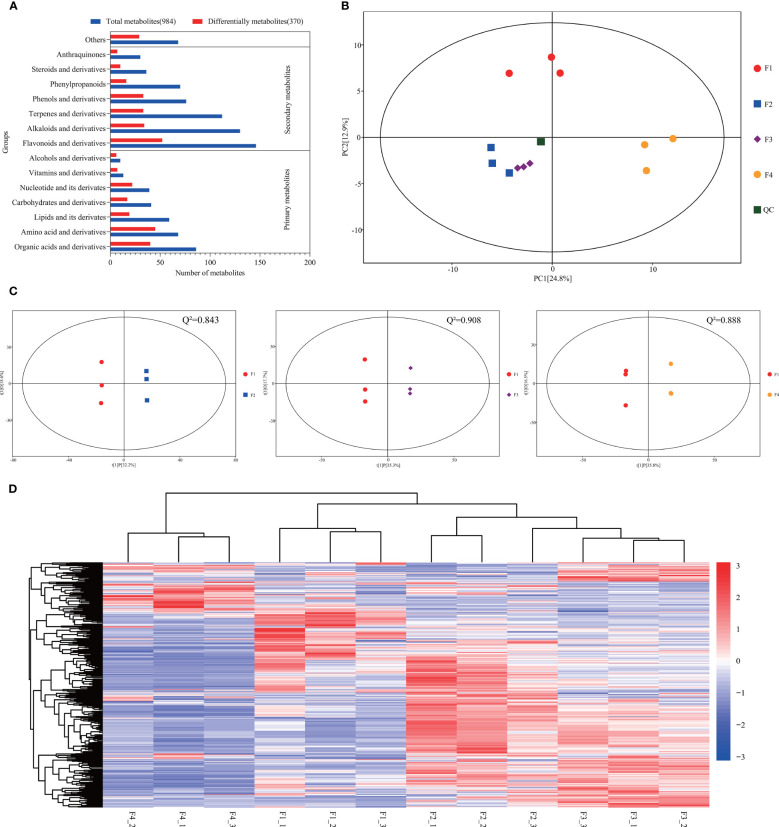
Metabolomic profiles during *P. cablin* flower development. **(A)** Classification of all the identified metabolites and DAMs. Red indicates the total identified metabolites, and blue indicates the DAMs. **(B)** PCA of the metabolite profiles of flower samples at four flowering stages. PC1 indicates the first principal component, and PC2 indicates the second principal component. **(C)** Score plot of the OPLS-DA of the metabolites at the four flowering stages. X axis, T score; Y axis, orthogonal T score. Q^2^ indicates the model predictability. **(D)** Bidirectional hierarchical clustering analysis of the DAMs and sample clustering analysis. The color palette represents the relative changes in metabolite contents among flower samples. The rows represent metabolites; the columns represent flower samples. Red and blue represent high gene expression and low gene expression, respectively. F1, inflorescence-bearing meristem stage; F2, flower bud stage; F3, full-bloom stage; F4, withered flower stage; and _1, _2, _3 represent three replicates.

Metabolic analysis showed that 984 metabolites were identified across all flower samples (**Table S8**). KEGG analysis showed that 916 metabolites were classified into 14 known classe (**Figure 6A**). The percentages of primary and secondary metabolites were 32% and 61%, respectively. A total of 370 DAMs (VIP > 1 and p value < 0.05) were identified (**Table S9**). Amino acids, organic acids, and their derivatives were the major primary DAMs, while the most abundant secondary DAMs were flavonoids and their derivatives, followed by terpenes, alkaloids, phenols, and their derivatives (**Figure 6A**). PCA revealed that each group tended to cluster together, separated by PC1 (24.8%) and PC2 (12.9%) (**Figure 6B**). The composition of metabolites was different at the four flower development stages, proved by the Q^2^ values of the OPLS-DA (all greater than 0.8) (**Figure 6C**), and by hierarchical clustering analysis (**Figure 6D**). These results suggested a significant distinction in all three comparison sets.

### 3.7 Functions of DAMs

**Figure 7:**
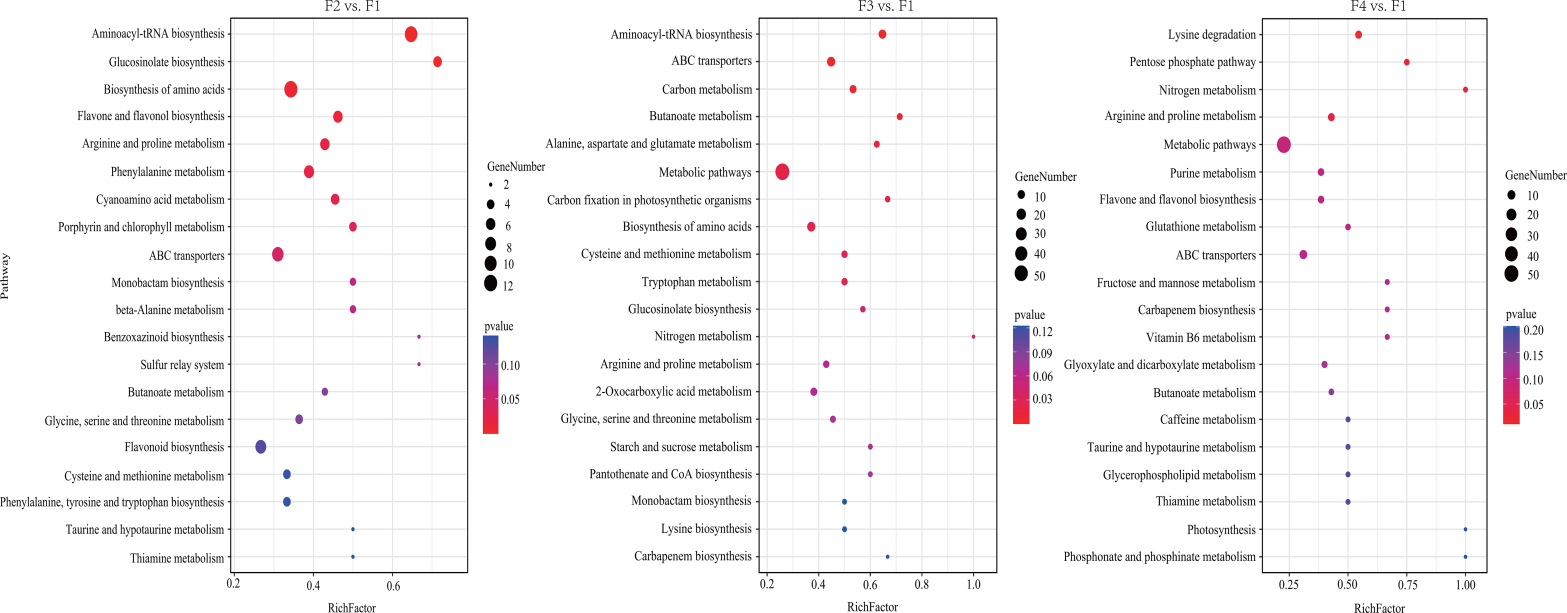
KEGG pathway enrichment analysis of DAMs at four flower stages of *P. cablin*. KEGG pathway enrichment analysis of DAMs in three comparison sets, including flower bud stage (F2) vs. inflorescence-bearing meristem stage (F1), full-bloom flower stage (F3) vs. inflorescence-bearing meristem stage (F1), and withered-flower stage (F4) vs. inflorescence meristem-stage (F1). The results are shown in the diagram with the twenty metabolic pathways along with the minimum p value. The bubble size represents the number of DAMs that were differentially accumulated in the pathway; the color range represents significant pathway enrichment. A p value < 0.05 and FDR < 0.05 were identified as significantly different metabolic pathways.

KEGG pathway enrichment analysis showed that 62, 75, and 68 DAMs were annotated, and 9, 12, and 4 KEGG pathways were significantly enriched in the three comparison sets (p < 0.05 and FDR < 0.05) (**Figure 7**; **Table S12**). Among them, amino acids such as lysine, arginine, and alanine was constantly active during *P. cablin* flower development. DAMs in two carbohydrate metabolism processes were significantly enriched at F3 and F4, while two significantly different metabolic pathways (flavone and flavonol biosynthesis, and porphyrin and chlorophyll metabolism) were enriched in the flower bud stage compared with the inflorescence-bearing meristem stage (**Figure 7**). These pathways were associated with flower color formation. This result might be attributed to the coloration of petals during flower organ development, and was generally consistent with the results from the KEGG pathway enrichment analyses of DEGs.

### 3.8 Accumulation patterns of predominant DAMs

**Figure 8:**
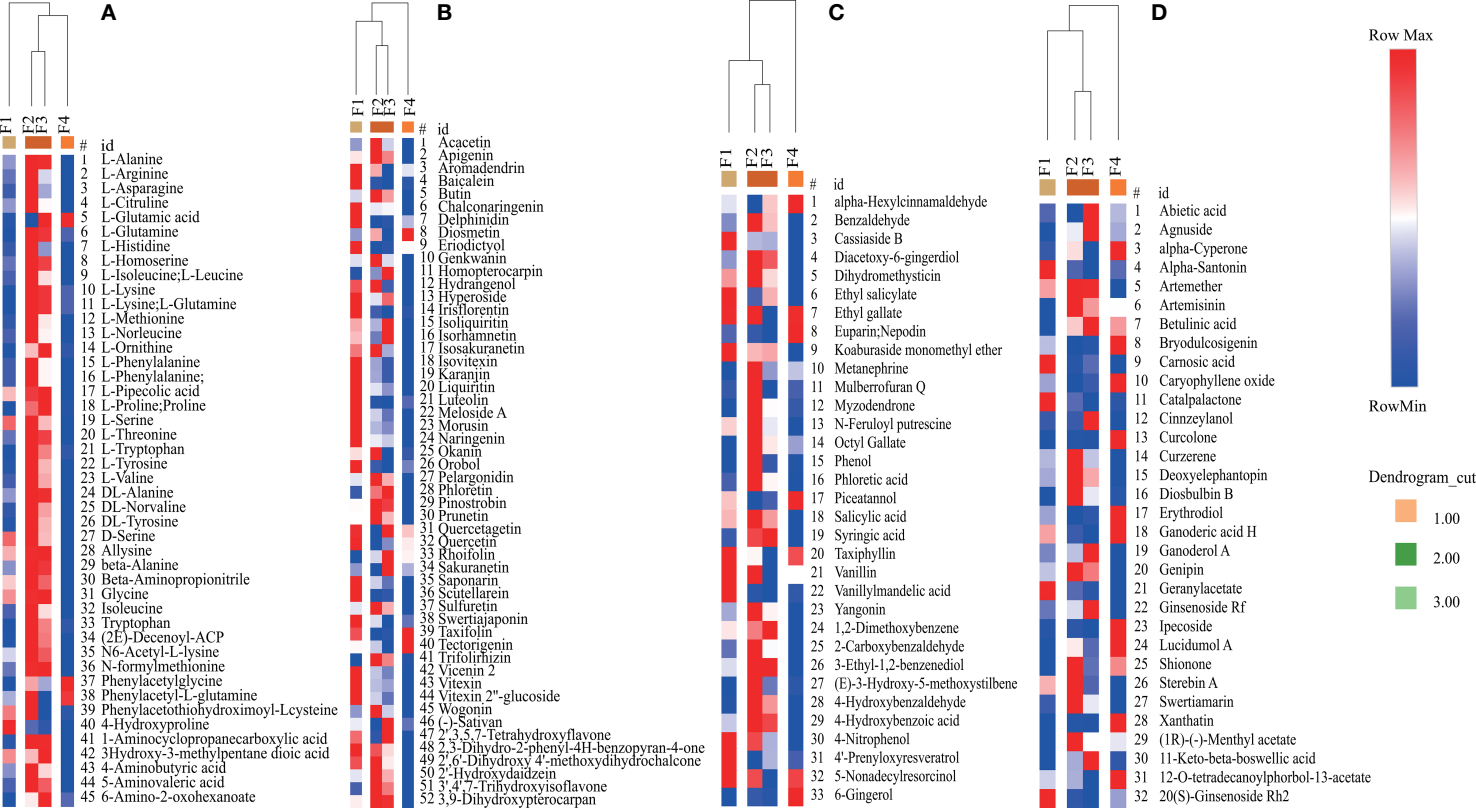
Relative changes in the contents of predominant DAMs during *P. cablin* flower development. **(A)** Relative changes in the contents of amino acids and their derivatives. **(B)** Relative changes in the contents of flavonoids and their derivatives. **(C)** Relative changes in the contents of phenols and their derivatives. **(D)** Relative changes in the contents of terpenes and their derivatives. The columns represent flower samples. Red and blue indicate the relative content and low relative content of metabolites, respectively. F1, inflorescence-bearing meristem stage; F2, flower bud stage; F3, full-bloom stage; and F4, withered-flower stage.

The accumulation patterns of the predominant DAMs (VIP>1 and p <0.05), including amino acids, flavonoids, terpenes, phenols, and their derivatives, were determined (**Figure 8**; **Table S13**). The Clustering analysis revealed that four predominant DAMs were clustered into three groups: group 1 (F1), group 2 (F2 and F3), and group 3 (F4) (**Figure 8**). These results were consistent with the results of PCA (**Figure 6B**). Twenty-two, four, and six amino acids were accumulated the highest at the F2 and F3 stages, the F4 stage, and the F2 stage, respectively (**Figure 8A**). Twenty-five, twenty-four, and three flavonoids and their derivatives exhibited the highest accumulation in group 1, group 2, and group 3, respectively (**Figure 8B**). In addition, twenty-one phenols and their derivatives exhibited the highest accumulation in group 2. Only five phenols and their derivatives and ten terpenes and their derivatives displayed the highest accumulation in group 3 (**Figures 8C, D**). Thus, the accumulation patterns of predominant DAMs were closely associated with flower development stage in *P. cablin*.

### 3.9 Coexpression network of flower development-related genes

To understand the possible regulatory relationships of flower development-related genes, a coexpression analysis was performed using the auxin, GA, MIKC-MADS, and aging-related DEGs (**Table S14**). Two independent modules (blue and turquoise) with high correlation coefficients (R^2^ > 0.85), were defined as clusters of highly interconnected genes (**Figure S5**). The results showed that the blue and turquoise modules were each correlated with F1, and F2 and F3, and contained 42 genes and 101 genes, respectively (**Figure S5C**, **Table S14**). The blue module consisted of 13 MIKC-MADS genes, 1 *FT* gene, 1 *FLK* gene, 10 *SPL* genes, 5 auxin signaling-related genes and 12 GA signaling-related genes. However, in the turquoise module, MADS genes comprised the greatest percentage (52.47%) of the genes, followed by GA signaling-related genes (25.74%) and auxin signaling-related genes (19.80%).

**Figure 9:**
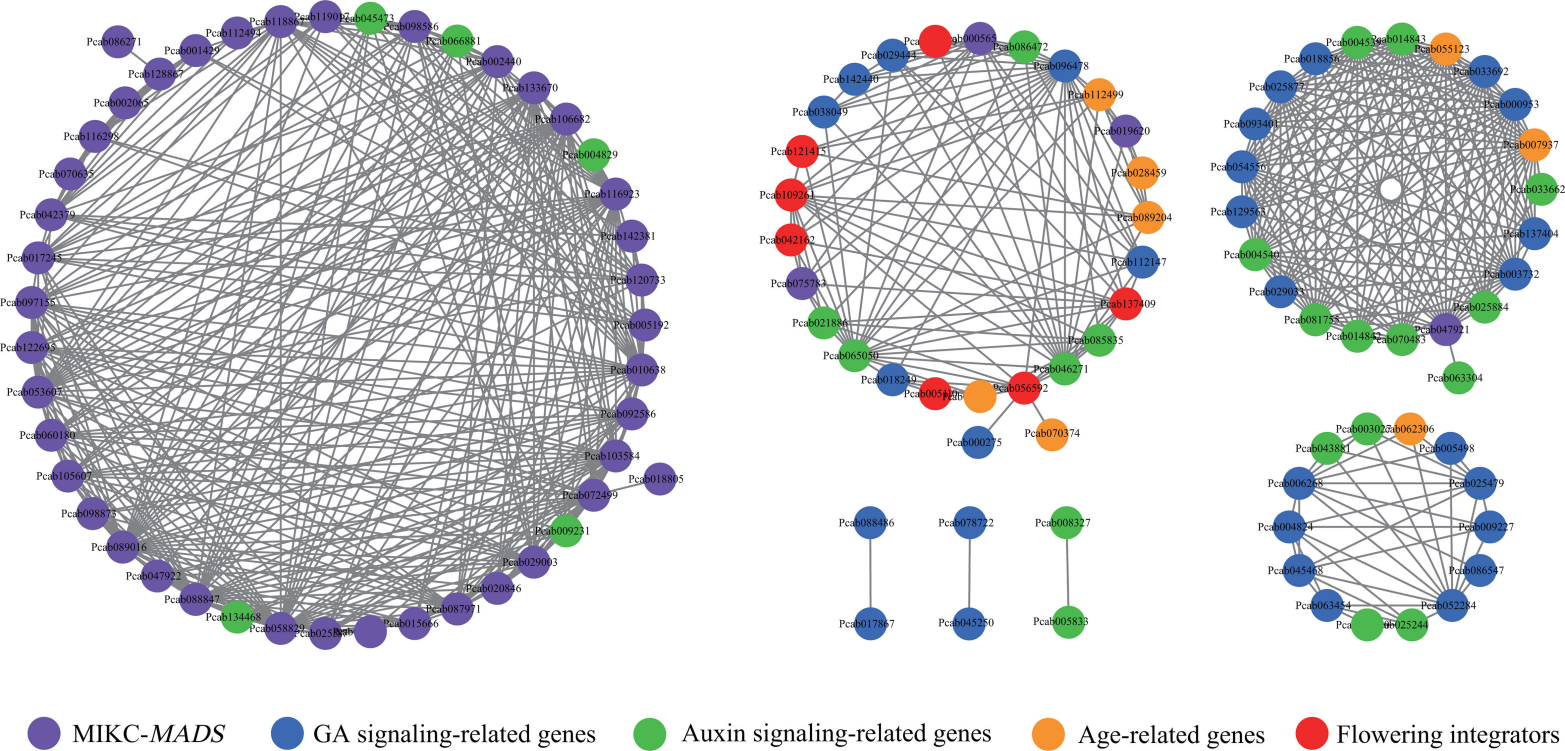
Coexpression network of flower development-related genes. The network was visualized using Cytoscapes. The purple, red, blue, green and orange round nodes represent MIKC-MADS genes, flowering integrators, GA signaling-related genes, auxin signaling-related genes, age-related genes, respectively. A TOM value > 0.4 is shown in the network plot.

The coexpression network derived from all modules showed that the possible regulatory relationship of MIKC-MADS genes with other genes was complicated (**Figure 9**). For example, *SVP-like* genes were correlated with *SOC1*, *GA2ox*, and *KAO*, and the TOM value ranged from 0.40 to 0.52 (**Table S14**). Two unknown MIKC-MADS genes (Pcab075783 and Pcab000565) were correlated with *SOC1*, *GA2ox*, *IAA*, and *GH3*. Moreover, *AG* (Pcab047921) was shown to be correlated with *SPL8*, *IAA*, *GA2ox*, *GA20ox*, and *GASA*, while *FT* was correlated with the *SVP-like* gene, *SPL7* and *KAO*. *SOC1* was strongly correlated with *SVP-like* genes, *GH3*, and *GA2ox* (**Table S14**).

The auxin and GA pathways are important hormone signaling pathways in flowering. Among auxin signaling-related genes, *IAAs* (Pcab004829 and Pcab045473) were strongly correlated with *AP3*, *SEP*, *AG* and *AP3*, respectively, while *AUX* (Pcab134468) was strongly correlated with *SEP* (**Table S14**). In the GA pathways, *SVP-like* genes (Pcab056592 and Pcab109261) were correlated with *GA2ox* and *KAO*. *AG* (Pcab047921) was correlated with *GA2ox*, *GA20ox*, and *GASA*. *GID* (Pcab137404) was strongly correlated with *GASA*. Notably, auxin signaling-related genes were strongly correlated with GA signaling-related genes. For example, *ARF* (Pcab043881) and *AUX* (Pcab003027) were strongly correlated with *GASA*. *IAA* and *SAUR* were strongly correlated with *GA2ox*, *GA20ox*, and *GASA* (**Table S14**). SPLs in aging pathway are important TFs involved in plant flowering. *SPL12* was correlated with *SOC1*, *SVP-like*, *GA2ox*, *GH3*, and *IAA*. *SPL8* was strongly correlated with *IAA* and *GASA*, while *SPL7* was strongly correlated with *SVP-like* (**Figure 9**; **Table S14**).

These results indicated that the GA, auxin, and aging pathways might form a cross-network to regulate the development of *P. cablin* flowers, and auxin signaling might participate in *P. cablin* flower development by affecting GA signaling.

### 3.10 Correlation analysis between the transcriptome and metabolome

Spearman’s rank correlation analysis was performed to evaluate the correlation coefficients between DEGs and DAMs during flower development (**Table S15**; **Figures S6**, **10**). Notably, 7801, 11245 and 10017 significantly correlated variables were involved in the biosynthesis and metabolism of primary and secondary DAMs at F2 vs. F1, F3 vs. F1 and F4 vs. F1, respectively (**Table S15**). The flavonoid biosynthesis (ko00941) and flavone and flavonol biosynthesis (ko00944) were the most significant metabolic pathways during flower development (**Figure S7**). Furthermore, the correlation network of DEGs and DAMs showed that many genes were positively correlated with flavonoids and phenylpropanoids (**Figure S8**; **Table S15**). For example, *4CL* was positively correlated with 9 flavonoids and 1 phenylpropanoid (imperatorin), while *GA3ox*, *GA20ox* and *GA2ox* were negatively correlated with 27 flavonoids and 5 phenylpropanoids. *IAA*, *ARF*, *AUX1/LAX*, and *SAUR* were also negatively correlated with 33 flavonoids. These results indicated that these genes related to GA and auxin signaling might be involved in the metabolism and biosynthesis of flavonoids, phenylpropanoids and their derivatives.

**Figure 10:**
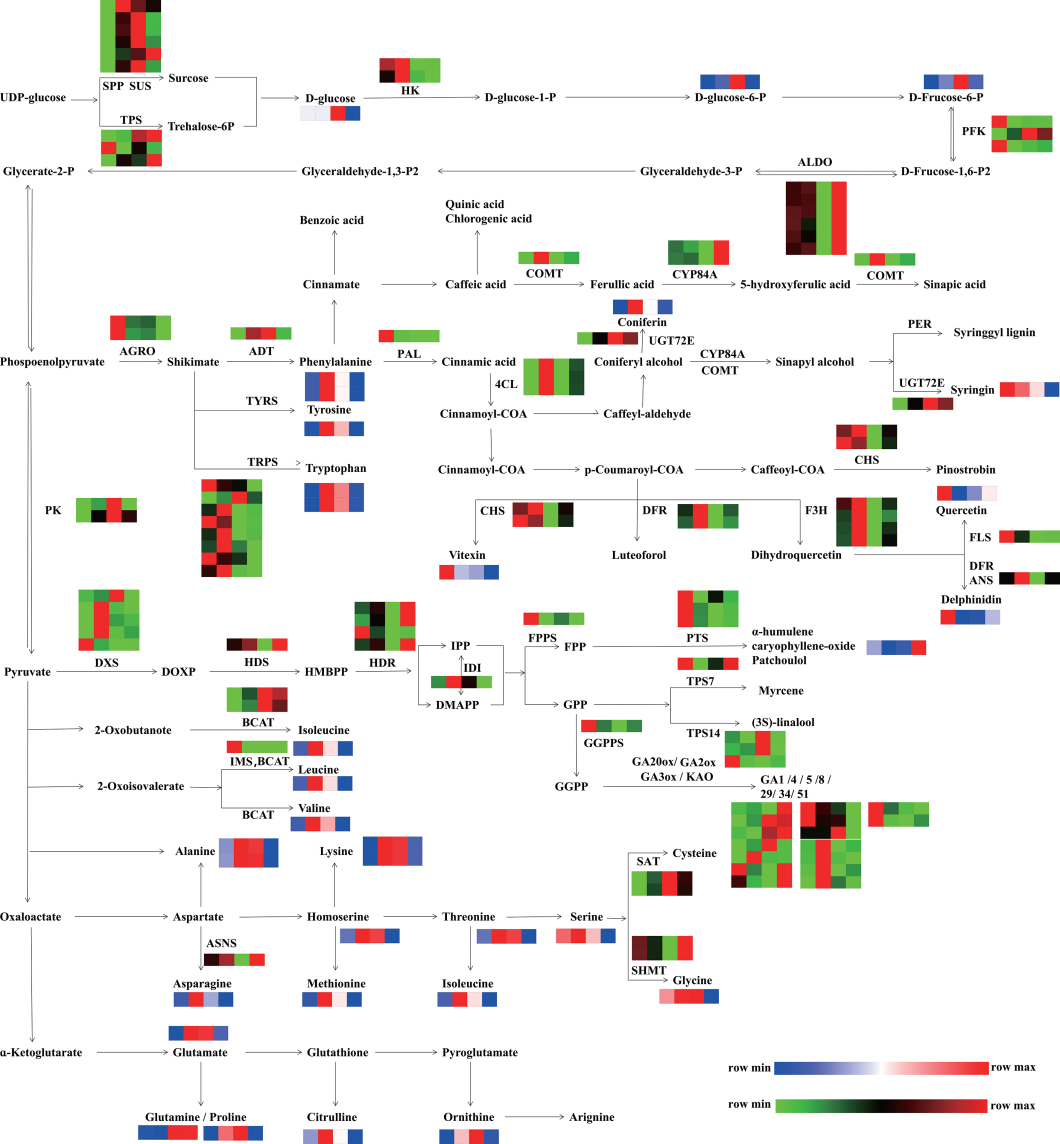
Integrated pathways of DEGs and DAMs involved in primary metabolism (starch and sucrose metabolism, carbon metabolism, biosynthesis of amino acids) and secondary metabolism (terpene biosynthesis, phenylpropanoid, phenols, and flavonoid biosynthesis). The pathway was redrawn based on the ko00500, ko01200, ko01230, ko00900, ko00904, ko00909, ko00940, ko00941, and ko00944 in the KEGG database (https://www.kegg.jp/). The red and green colors indicate upregulated unigenes and downregulated unigenes, respectively. The red and blue colors indicate the relative content and low relative content of metabolites, respectively. From left to right, the four squares represent the following stages: F1, inflorescence-bearing meristem stage; F2, flower bud stage; F3, full-bloom stage; and F4, withered-flower stage, respectively. SPP, sucrose-phosphatase; SUS, sucrose synthase; TPS, trehalose-phosphate phosphatase; HK, hexokinase; PFK, 6-phosphofructokinase 1; ALDO, fructose-bisphosphate aldolase, class I; PK, pyruvate kinase; AGRO, phosphoheptulonate synthase; PAL, phenylalanine ammonia-lyase; BCAT, branched-chain amino acid aminotransferase; TRPS, tryptophan synthase; 4CL, 4-coumarate CoA ligase**;** ASNS, aspartate-ammonia ligase; CYSE, serine O-acetyltransferase; ADT, arogenate dehydratase; IMS, 2-isopropylmalate synthase; UGT72E, coniferyl-alcohol glucosyltransferase; DFR, flavanone 4-reductase; ANS, anthocyanidin synthase; PER, peroxidase; CHS, Chalcone synthase; FLS, flavonol synthase; COMT, caffeic acid 3-O-methyltransferase; F3H, flavanone 3-dioxygenase; SAT, serine acetyltransferase; SHMT, glycine hydroxymethyl transferase; FPPS, farnesyl pyrophosphate synthase; GGPPS, geranylgeranyl diphosphate synthase; IDI, isopentenyl pyrophosphate isomerase, HDR, 1-hydroxy-2-methyl- 2-butenyl 4-diphosphate reductase; HDS, 2-C-methyl-D-erythritol 2,4-cyclodiphosphate synthase; DXS, 1-deoxy-D-xylulose-5-phosphate synthase; PTS, patchoulol synthase; TPS7, trans-alpha-bergamotene synthase*-*like; TPS14, S-linalool synthase; KAO, ent-kaurenoic acid oxidase; GA3ox, GA 3-oxidase; GA2ox, GA 2-oxidase.

To illustrate the correlations of DEGs and DAMs related to carbohydrates, phenylpropanoids, flavonoids and phenols, and amino acids, an integrated pathway at four stages was constructed (**Figure 10**). In the starch and sucrose metabolism section, the accumulation of D-glucose, D-glucose-6-P, and D-fructose-6-P increased from the F1 to F3 stage and then decreased at the F4 stage, which was similar with the expression of the pyruvate kinase (*PK*). In the phenylpropanoids, flavonoid, and phenol biosynthesis pathway, *ADT* was highly expressed from the F2 to F3 stage, fitting the accumulation of phenylalanine. The expression levels of *CHS*, *FLS* and *ANS* were high at the F1 and F2 stages, while the transcription levels of *4CL*, *DFR*, and *F3H* were highest at F3. The accumulation patterns of their products (vitexin, quercetin, delphinidin, and luteoforol) were highly consistent with the gene expression patterns. In amino acid biosynthesis, the expression pattern of *ASNS* was consistent with the accumulation of asparagine. The accumulation of alanine, lysine, homoserine, methionine, threonine, leucine, valine, isoleucine, glutamate, citrulline, and ornithine increased from the F2 to F3 stage. These results indicated that the active metabolism of starch and sucrose might be conducive to the biosynthesis of phenylpropanoids, flavonoids, and amino acids.

## 4 Discussion

### 4.1 Effects of flower development on the main active constituents in *P. cablin*

*P. cablin* has been widely cultivated due to the high demand for its essential oils, a crucial industrial ingredient in medicine, perfumes and cosmetics. Besides leaves, the inflorescences of *P. cablin* also accumulate large amounts of oil, which are composed of 17 sesquiterpenes (97.7%), the main active ingredient of patchoulol (Verma et al., 2019). In our results, 111 terpenoids and their derivatives were identified across the four flower stages and the relative content of sesquiterpenoids was highest (82.77%-88.80%) (**Table S16**). Recent studies of lavender have shown that terpenoid contents fluctuated with flower development stage (Li et al., 2019). Similarly, our results showed that 32 differentially accumulated terpenoids and their derivatives were identified, and their accumulation patterns were related to the flower development stage. We found that, patchoulol, a key ingredient in assessing the quality of *P. cablin*, was the predominant sesquiterpene present at each flower stage, and its relative content changed little during flower development (**Figure S9**). In addition, the relative content of patchoulol in the leaves of flowering and nonflowering plants also changed slightly (**Figure S9**).

### 4.2 Energy consumption during *P. cablin* reproduction

As flower development progresses, flowers shifts from autotrophy to heterotrophy, accompanied with a progressively decreased photosynthesis rate and the emergence of anther secondary metabolism (Müller et al., 2010; Muhlemann et al., 2012). As expected, the pathways involved in the biosynthesis of secondary metabolites were significantly enriched from the bud stage to the withered flower stage (**Figure 2**). The accumulation of secondary metabolites were related to color, scent and taste (Ruan et al., 2010; Shan et al., 2019), depended on the pollinator, precursors and energy generated from the metabolism of primary metabolites (Payyavula et al., 2013; Borghi and Fernie, 2017). Starch and sucrose metabolism is generally active (**Figure 2**) (Jing et al., 2020; Lu et al., 2020; Cai et al., 2021), and participates in the biosynthesis of flavonoids and phenylpropanoids during flower development (Ferri et al., 2011; Payyavula et al., 2013). Sucrose, a major type of carbohydrates, is translocated from photosynthetically active tissues to nonphotosynthetic sinks (flowers) (Eveland and Jackson, 2012; Borghi and Fernie, 2017), and is degraded rapidly to provide carbon skeleton and energy for secondary metabolites (Eveland and Jackson, 2012). According to the KEGG enrichment results, a number of genes involved in phenylpropanoids, alpha-linolenic acid metabolism, and starch and sucrose metabolism, significantly increased from the F2 to F3 stage and then decreased at the F4 stage (**Figure 2**, **Table S4**). Alpha-linolenic acid metabolism is closely linked to JA (jasmonic acid) synthesis, which influence the various metabolites production, such as terpenoids and pyrethrin *via* the MEP pathway (Ghorbel et al., 2021; Zeng et al., 2022). Moreover, in the integration pathway of DEGs and DAMs (**Figure 10**), the starch and sucrose might be important energy sources for *P. cablin* reproduction, and phenylpropanoids, flavonoids, and amino acids might accumulate due to activated starch and sucrose metabolism. These results were similar to those from a previous study of *Lonicera japonica* Thunb. flower development (Yang et al., 2019).

In flower development, amino acids play a crucial role in enzyme synthesis, osmotic regulation, and providing nitrogen and energy for pollen and ovule maturation (Biancucci et al., 2015; Gaufichon et al., 2017). Recently, researchers discovered that the contents of amino acids increased in *Agave amica* flowers at the bud and full-bloom stages (Kutty et al., 2021). Interestingly, we also detected increased accumulation of 53 differentially accumulated amino acids at the bud and full-bloom stages, followed by a decrease at the withered flower stages. Flower opening requires osmotic oscillations including amino acids, which is followed by an influx of water into cells (Forterre, 2013; van Doorn et al., 2013). This finding might explain why amino acids accumulated mainly at the bud and full-bloom stages. In addition, our data showed that many amino acid metabolism and degradation pathways were enriched at the withered flower stage. The reason might be decomposition and translocation of proteins from senescent flowers to phloem (van Doorn, 2004). Therefore, these results suggested that the energy and carbon sources consumed during *P. cablin* reproduction might be derived from sucrose and starch metabolism, whereas amino acids are used more as structural components to maintain the proper status and shape of flowers. In addition, phenylpropanoids, flavonoids and amino acids might accumulate due to activated starch and sucrose metabolism.

### 4.3 *P. cablin* flower pigment formation

Chlorophyll, carotenoids, and anthocyanins contributes to various flowers color, which are also affected by copigmentation, vacuolar pH, metal chelation and other processes (Trouillas et al., 2016; Stavenga et al., 2021). In our research, anthocyanin-containing compound biosynthesis processes were significantly more active from F2 to F4, indicating that anthocyanins are related to the color formation of *P. cablin* flowers. Copigmentation is a natural approach to stabilize anthocyanins, by forming noncovalent complexes (Stavenga et al., 2021). Flavonoids and phenolic acids, stabilizing anthocyanins and intensify color, are the most effective copigments (Klisurova et al., 2019). These two classes of metabolites are synthesized from the phenylpropanoid pathway (Buer et al., 2010). Previous research has found that flavonoids and phenylpropanoids-related genes tend to be highly expressed during the flower color formation stage (Zheng et al., 2019). In our study, the phenylpropanoid and flavonoid biosynthesis pathways were also enriched from the bud to withered flower stages in *P. cablin* (**Figure 2**; **Table S4**). Our results were generally consistent with the studies on *Rosmarinus officinalis* flowers (del Baño et al., 2003).

Previous research has shown that the contents of phenolic compounds and flavonoids change during flower development in some rose species (Elmastaş et al., 2017; Chamani et al., 2020). In the present study, flavonoid and phenolic acid contents also changed during the development of *P. cablin* flowers, indicating similar accumulation patterns between the bud and full-bloom stages. The spikelets of *P. cablin* have whorls of flowers ranging in color from white to lilac, purplish-white petals, purple stamen filaments, yellow anthers, and a green calyx and ovaries. Buds and full-bloom flowers contain similar floral organs, including purplish-white petals, purple stamen filaments, and yellow anthers, which are different from those at other stages. This finding may explain why the accumulation of metabolites at the bud and full-bloom stages was similar. Taken together, our results suggested that flavonoids and phenolic acids might contribute to pigment formation in *P. cablin*.

### 4.4 GA and auxin signals associated with *P. cablin* flower development

Plant hormones, such as GAs, auxin, and cytokinin, have important roles in flowering (Conti, 2017). Among them, GAs play key roles in plant flowering (Mutasa-Gottgens and Hedden, 2009; Conti, 2017). First, GAs can accelerate floral induction in several species, such as *A. thaliana* (Sven Eriksson et al., 2006) and *Eriobotrya japonica* (Jing et al., 2020). Second, GAs can promote the growth and maturation of floral organs, such as petals (Plackett et al., 2012), stamens (Plackett et al., 2011), ovaries (Lange and Lange, 2016), anthers and pistils (Hu et al., 2008). GAs are synthesized in anthers and pistils, and transfer to other floral organs for the development of them (Pimenta Lange et al., 2012; Binenbaum et al., 2018). The biosynthesis of active GAs involves 12 steps, regulated by a series of enzymes (**Figure 3A**) (Fan et al., 2017). As the starting and final enzymes, GA3ox and GA20ox are particularly important for controlling bioactive GA levels. In our study, the highest expression of *GA3ox* was detected at the flower bud stage while *GA20ox* was mainly upregulated from the full-bloom to the withered flower stages (**Figure 3B**). A recent survey found that *VpGA3ox* and *VpGA20ox* participate in the initiation of floral organ formation in *Viola philippica*, proved by upregulated expressions in the primordia of pistils and stamens (Li et al., 2021). Therefore, GA signaling might participate in floral organ formation and maturation during *P. cablin* flower development.

GA signaling exerted opposite effects on different flower development stages (Yamaguchi et al., 2014), since the concentration of GAs is relatively high during the flower bud stage and then decreases after anthesis (Cheng et al., 2004; Serrani et al., 2007; Iftikhar et al., 2020). A recent study of *Petunia hybrida* also found that GA1 and GA4 were detected at negligible amounts when the phenylpropanoid volatiles were actively produced and emitted after anthesis, implying a negative regulation of GAs on the synthesis of phenylpropanoids (Ravid et al., 2017). Similarly, no corresponding products were detected in our results, although several key enzyme genes (*KAO*, *GA2ox*, *GA3ox*, and *GA20ox*) were active at the flower bud and full-bloom stages. In *A. thaliana* flowers, two potential sites (stamens and receptacles) for bioactive GA synthesis were identified, suggesting that GAs might be dynamically transported from these organs to other parts during flower development (Hu et al., 2008). Thus, we proposed that GAs may have been transferred to other parts of the inflorescence to produce scent-associated metabolites during the development of *P. cablin* flowers. These results also suggested that GA signaling might be a regulatory switch between flower developmental and flower-related scent metabolism in *P. cablin*.

Auxin is another important hormone in the flowering process, including inflorescence and floral organ initiation (Cucinotta et al., 2021). Auxin signal transduction involves four core components: AUX/IAA, AUX/LAX, transport inhibitor response 1/auxin signaling F-box protein (TIR1/AFB), and ARF family member proteins (Swarup and Péret, 2012; Lavy and Estelle, 2016). As shown in our results, auxin signal transduction was clearly enriched at the bud flower stage (**Figure S1**). Interestingly, all *AUX*/*LAXs*, major auxin inflow carriers, were mainly upregulated from the bud to withered flower stages (**Figure S2**), indicating that high concentration of auxin is required for *P. cablin* flower development. In nature, IAA presents in active free-state or inactive combined-state, controlled by GH3 (Ludwig-Müller, 2011). In our study, expressions of most *IAA* genes were upregulated from the bud stage to the full-bloom stage, however the transcript levels of *GH3* genes decreased at same stages (**Figure S2**), suggesting that more active auxin are needed for the continuous stimulation of *P. cablin* flower development.

Research has shown that auxin participates in flower closing-opening activity, proved by upregulated expressions of *IAA* and *ARF* during flower opening but downregulated expressions during closing (Ke et al., 2018). Similarly, our transcriptomic data showed that many homologs of *IAA*, *ARF*, and *SAUR* were maintained high transcript levels from the bud to the full-bloom stage (**Figure S2**). According to these results, the activation of auxin signaling at the full-bloom stage might be associated with flower opening and the maintenance of blooms. In addition, auxin signaling has many functions in the growth of stamens, petals, and pedicels (Cheng et al., 2006; van Doorn et al., 2013) and accelerates the maturation and development of pollen, anthers and ovules (Cecchetti et al., 2008; Galbiati et al., 2013). According to our observations, the floral organs within most bud samples of *P. cablin* were incomplete and still developing. Therefore, the activation of auxin signaling at the bud flower stage might favor floral organ formation.

In summary, GA and auxin signaling might be involved in floral organ development in *P. cablin*. In addition, GA signaling might be a regulatory switch between flower development and flower-related scent/color metabolism, while auxin signaling might be beneficial for flower opening.

### 4.5 Potential regulatory relationships of flower-related genes

Three categories of genes are responsible for flower development, including floral pathway integrators (*FT* and *SOC1*), floral meristem identity genes (*AP1* and *FUL*) and floral organ identity genes (*AP2*, *AP3*, *PI*, *SEP* and *AG*) (Satish and Manju, 2018; Cao et al., 2021). Floral organ identity genes are regulated by flower pathway integrators. For example, *AtFT* can positively regulate the expression of *AtAP1* and *AtFUL*, and *AtSOC1* upregulates the transcription of *AtAP2*, *AtPI*, and *AtAG via AtLFY* (Blázquez, 2000). As expected, our results showed that *FT* was correlated with *SVP-like*, *SPL7*, and *KAO*, whereas *SOC1* were strongly correlated with the *SVP-like* and two new MIKC-MADS genes. Another function of flowering integrators is to integrate the environmental and internal flower induction signals. For example, *AtSPL3/4/5* and *AtSPL2/9/10/11/13/15* can directly bind to *AtFT and AtSOC1* in the shoot apex meristem, and then induce the expression of *AtAP1*, *AtLFY*, and *AtFUL* (Xu et al., 2016). *EjSPL3/4/5* can significantly upregulate the expression levels of *EjSOC1-1*, *EjAP1*, and *EjLFY-1* in *E. japonicat* (Jiang et al., 2019). *PvSPL* also directly upregulates the expression levels of *PvSEP3* and *PvAP1*/*FUL* to promote *Panicum virgatum* flowering (Yamaguchi et al., 2009; Gou et al., 2019). According to our results, *SPL12* was correlated with *SOC1*, and *SVP-like* gene, whereas *SPL7*, *SPL3*, and *SPL8* were strongly correlated with *SVP-like*, new MIKC-MADS gene (Pcab019620), and *AG* genes. This result suggested that the transcription of *SOC1*, *FT*, *SVP*, *AG* and new MICK-MADS genes might be regulated by aging-related genes (*SPL*) in *P. cablin*.

Hormone signaling pathways are important for flower development. Previous reporters also revealed that GAs regulate the transcription of *AtSVP* and *AtSOC1 via* an *AtDELLA*-mediated signaling pathway (Li et al., 2008). Interestingly, *AtSVP* negatively regulates GA signaling by repressing the transcription of *GA20ox* (Leijten et al., 2018). In our results, *SVP-like* genes were correlated with *GA2ox* and *KAO*, while new MIKC-MADS genes and *AG* were correlated with *GA2ox*, respectively. Auxin signaling appears to be a positive regulator of the flowering process (Renau-Morata et al., 2021). *AtARF5* is presumed to be closely associated with the initiation of flower primordia and organs, and significantly upregulates the expression level of *AtLFY* (Krizek and Eaddy, 2012; Yamaguchi et al., 2016). In our results, *IAA* was strongly correlated with *AP3*, *SEP*, *AG*, and *GH3*, while *AUX* was strongly correlated with *SEP*. These results also indicated that auxin signaling might regulate *P. cablin* flower development by affecting the expression of these flower development-related genes. In addition, auxin can promote flowering by regulating the GA content and promoting DELLA degradation (O’Neill and Ross, 2002; Fu and Harberd, 2003; Frigerio et al., 2006). Similarly, our results also showed that auxin signaling-related genes were strongly correlated with GA signaling-related genes (**Figure 9**). These results implied that GA and auxin signaling might regulate *P. cablin* flower development in a cross-linked manner.

The flower induction regulation pathway typically forms a regulatory network integrating flower stimulation and triggering the transition from vegetative to reproductive stages. Previous research found that the aging-related *AtSPL3/9/15* gene interacts with AtDELLA and impairs the activation of flower-related genes (such as *AtSOC1*), suggesting that *SPLs* are key targets of GA signaling (Yu et al., 2012; Hyun et al., 2017; Li et al., 2018). In our results, *SPL12* was correlated with *GA2ox*, *GH3*, and *IAA*, while *SPL8* was strongly correlated with *IAA* and *GASA* (**Figure 9**). These results suggested crosstalk between GA, auxin, and aging pathways during flower development in *P.cablin*. In summary, the GA, auxin, and aging pathways might form a cross-regulatory network to regulate *P. cablin* flowering.

### 4.6 GA and auxin signaling might participate in the synthesis of phenylpropanoids and flavonoids during flower development

Plants also produce volatiles and pigments, including phenylpropanoids and flavonoids, for pollinators during flowering. Those natural products are regulated by core proteins, including PAL, 4CL, C4H, and PAAS. Hormone signals are an important regulatory link in floral color/scent during flower development (Ravid et al., 2017; Lu et al., 2020). In our results, the phenylpropanoid and flavonoid biosynthesis pathways were also enriched from the bud to withered flower stages in *P. cablin* (**Figure 2**; **Table S4**), and GA signaling-related genes (*GA3ox*, *GA20ox* and *GA2ox*) were negatively correlated with 27 flavonoids, 5 phenylpropanoids and 8 terpenes. Auxin signaling-related genes (*IAA*, *ARF*, *AUX1*, *LAX*, and *SAUR*) were also negatively correlated with 33 flavonoids, 7 phenylpropanoids, and 15 terpenes (**Figure S8**). These results suggested GA and auxin signaling might be involved in the metabolism and biosynthesis of flavonoids, phenylpropanoids, terpenes and their derivatives. In addition, DELLA proteins can bind with a variety of transcription factors (i.e., AtSPL15, AtMYC2, and AtMYB23) and prevent them from binding to their target genes (Qi et al., 2014; Davière and Achard, 2016). EOBI, EOBII and ODO1 in the MYB family, were proven to regulate the expression of some phenylpropanoid-related genes (*CS*, *CM*, and *PAL*) (Verdonk et al., 2005; Spitzer-Rimon et al., 2010). What’s more, DELLA can also bind with AtMYC2 to inhibit the expression of sesquiterpene synthase genes (*AtTPS11* and *AtTSP22*) (Hong et al., 2012). Similarly, PatMYC2b1/PatMYC2b2 can directly regulate the expression of *PatPTS* in *P. cablin* (Wang et al., 2019). Thus, we propose that DELLA might bind to some MYB and MYC TFs to regulate some genes related to the phenylpropanoid, flavonoid and terpene biosynthesis pathways.

**Figure 11:**
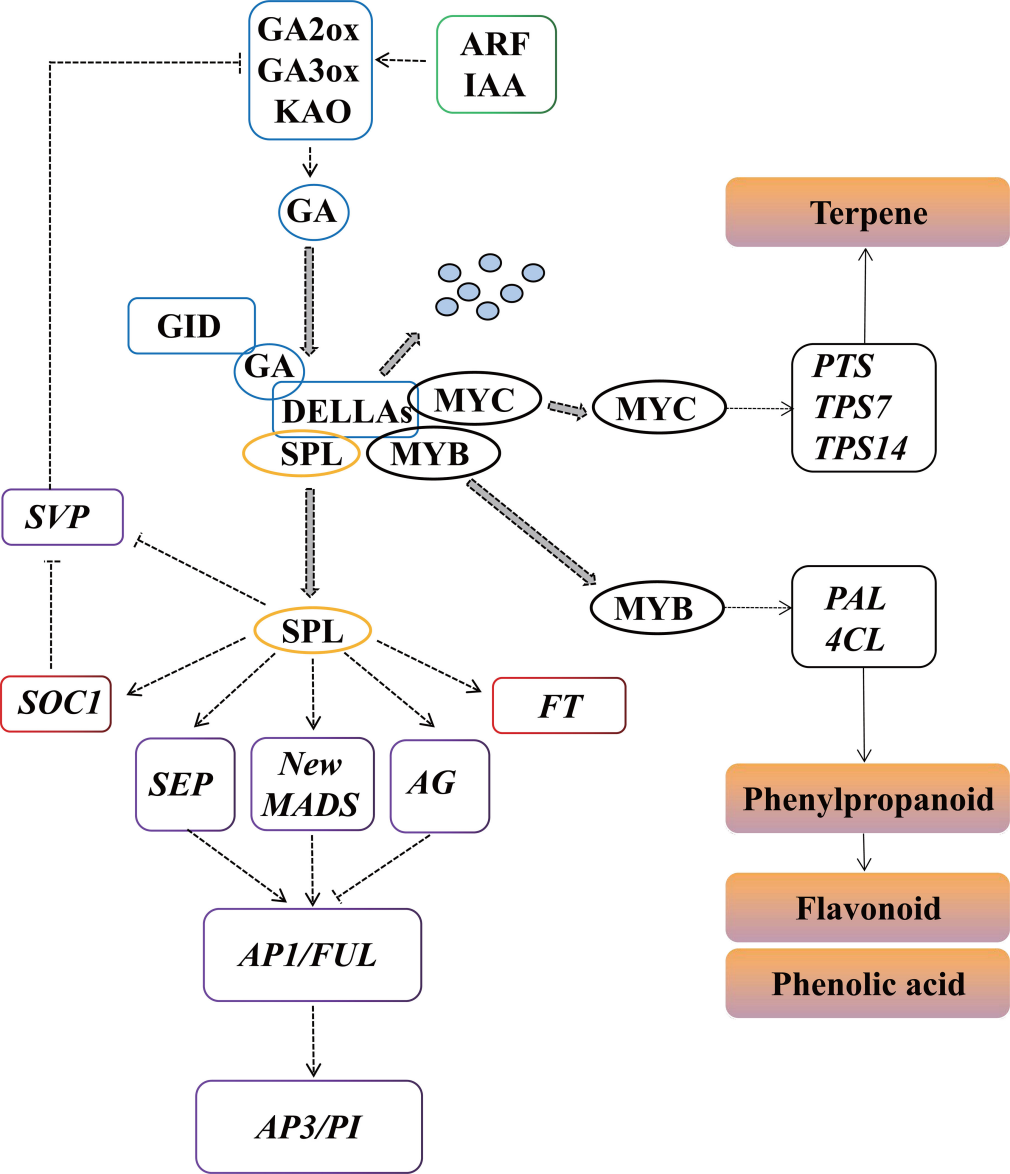
Predicted regulatory model involved in *P. cablin* flower development. KAO, ent-kaurenoic acid oxidase; GA3ox, GA 3-oxidase; GA2ox, GA 2-oxidase; GID1, gibberellin receptor; IAA, indole-3-acetic acid; ARF, auxin response factor; GH3, Gretchen Hagen3; SPL, squamosa promoter binding protein*-*like; FT, FLOWERING LOCUS T; SOC1, SUPPRESSOR OF OVEREXPRESSION OF CONSTANS 1; AP1, APETALA1; FUL, FRUITFUL; PI, PISTILLATA; AP3, APETALA3; SEP, SEPALLATA; AG, AGAMOUS, PAL, phenylalanine ammonia-lyase; 4CL, 4-coumarate CoA ligase, PTS, patchoulol synthase; TPS7, trans-alpha-bergamotene synthase-like; TPS14, S-linalool synthase.

Based on our results, a proposed model of the molecular and genetic networks regulating *P. cablin* flower development is shown in **Figure 11**. GA signaling acts as an important regulatory switch connecting flower development and pollinator attraction. DELLAs are an important inhibitor of GA signaling and are degraded after interacting with the GA receptor GID1 when GA accumulates at high levels. IAAs can induce the expression of *GA2ox*, *GA3ox* and *GA20ox* to increase the biosynthesis of GAs, which results in DELLA degradation to promote flower development. SPL induces the expression of *SOC1*, *FT*, *SEP*, *AG*, *AP1/FUL* and new MIKC-MADS to promote flower development in *P. cablin*, and is inhibited by DELLA. In addition, the floral repressor *SVP*, another target of DELLA, negatively regulates GA signaling by repressing the transcription of *GA2ox*, *GA3ox* and *GA20ox*. Meanwhile, GA also negatively regulates the transcription of genes (*PAL* and *4CL, PTS and TPS*) involved in phenylpropanoid, flavonoid and terpene production *via* DELLA-mediated signaling pathway.

## 5 Conclusions

*P. cablin* is an important medicinal plant species known for its essential oil. However, due to the difficulty in flowering, it is mainly cultivated and preserved though cottage propagation. Cottage propagation tends to result in virus accumulation and quality losses, hindering the development and utilization of *P. cablin*. Flowering, the beginning of seed formation, is regulated by precise molecular and genetic networks involved in a variety of signaling pathways. A model of the molecular and genetic regulatory networks of *P. cablin* flower development is proposed in **Figure 11**. In this model, three signaling pathways (GA, auxin and aging) can regulate the transcript levels of floral organ identity genes (*AP1*, *AG*, *FUL*, *PI* and *SEP*, etc.) to promote the development of floral organs and anthesis. Moreover, GA also negatively regulate the transcription of some genes involved in floral color/scent (phenylpropanoid and flavonoids) production *via* the DELLA-mediated signaling pathway. GA signaling acts as an important regulatory switch connecting flower morphology and pollinator attraction, to maintain the flowers in the best pollination state for successful reproduction. These results will provide a basis for future studies on the sexual reproduction and breeding of *P. cablin*.

## Data availability statement

The datasets presented in this study can be found in online repositories. The names of the repository/repositories and accession number(s) can be found in the article/**Supplementary Material**.

## Author contributions

YW conceived, designed, and supervised the research project. CZ, XL and YL performed the experiments. CZ, JY and GY analyzed the data. CZ and YL wrote the manuscript. HY and DY provided input on the data presentation and critically revised the manuscript. All authors contributed to the article and approved the submitted version.
