# Supplementary material for: An integrated transcriptome and metabolome analysis reveals the gene network regulating flower development in Pogostemon cablin
Source: Front Plant Sci. 2023 Jun 29;14:1201486. doi: 10.3389/fpls.2023.1201486 (PMC10340533; doi:10.3389/fpls.2023.1201486)
Supplement: Supplementary file 1 [file DataSheet_1.pdf]

Supplementary Material

An integrated transcriptome and metabolome analysis reveals the gene network regulating flower development in *Pogostemon cablin*

Chan Zhang1†, Xiaofeng Liu1†, Ya Liu1†, Jing Yu1, Guanglong Yao1, Huageng Yang1, Dongmei Yang1, and Yougen Wu1*

†These authors contributed equally to this work and share first authorship

*** Correspondence:** Yougen Wu: wygeng2003@163.com

1 Supplementary Figures and Tables

1.1 Supplementary Figures

Figure S1. GO functional enrichment of DEGs in different flowering stages in *P. cablin*.

Figure S2. KEGG pathway and expression analysis emphasizing the phytohormone signaling transduction DEGs in *P. cablin*. KEGG analysis emphasized the differential gene expression of phytohormone signaling transduction. The pathway was redrawn based on ko04075 in the KEGG database (https://www.kegg.jp/). Gene expression level [$\log_2$(TPM+1)] patterns of related phytohormone responsive signaling transduction factors at the four flower stages. The red diamonds indicate differential expression genes.

Figure S3. Phylogenetic analyses of MADS **genes of *P. cablin* and *Arabidopsis thaliana*.** The red dots indicate differentially expressed MADS-box genes.

Figure S4. Heatmap of differentially expressed *SPL* genes at the four flower stages. Red indicates a high expression level and green indicates a low expression level.

Figure S5. Coexpression of flower development-related genes at the four stages. (A) Correlation analysis to identified the soft-cut value β. (B) Hierarchical cluster tree showing coexpression modules identified by WGCNA. Each leaf in the tree is one gene. The major tree branches constitute 2 modules labeled by different colors. (C) Epigengene network heatmap plot of the blue and turquoise modules.

Figure S6. Heatmap of the correlation among DEGs and DAMs by the Spearman's rank correlation coefficients at the four flower development stages. "*" ($p < 0.05$) represents a significant correlation.

Figure S7. The Flavonoid biosynthesis (ko00941) and flavone and flavonol biosynthesis (ko00944) pathway. Green and yellow indicate the up-regulated and down-regulated DEGs and DAMs, respectively.

Figure S8. Correlation network for the transcriptome and metabolome. Correlation coefficients > 0.8 or <-0.8 and $p < 0.05$ are displayed in the network plot. The network is involved in the biosynthesis and metabolism of flavonoids and phenylpropanoids based on the results of F2 vs.

F1 comparison set. Red and blue lines (edges) represent positive and negative correlations, respectively.

Figure S9. The relative content of patchoulol in flower and leaf of *P. cablin*. (A) The relative content of patchoulol in the four flower stages. (B) The relative content of patchoulol in leaves of flowering and non-flowering plants. (C) and (D) represents eic curve of flowers and leaves, respectively. "*" indicates significant differences, as determined using one-way ANOVA followed by Tukey's test ($p<0.05$).

1.2 Supplementary Tables

Table S1. Summary of RNA-seq data quality control.

Table S2. Annotation of DEGs with different databases.

Table S3. The significantly different metabolic pathways by GO analysis of the three comparison sets.

Table S4. The significantly different metabolic pathways by KEGG analysis of the three comparison sets.

Table S5. Summary of re-characterized genes annotated in pathways related to terpenoid biosynthesis.

Table S6. DEGs related to terpenoid biosynthesis.

Table S7. P-value and FDR of DEGs involved in terpene synthase, the MADS **Ts family and plant hormone signals.** The p-value and FDR were calculated by DEseq2 and one-way ANOVA and post-hoc tests. Adjusted p-value (FDR) cutoff: 0.05; Post-hoc analysis: Tukey test.

Table S8. Summary of metabolome profiling for *P. cablin*.

Table S9. Differential metabolites in flower development in *P. cablin*.

Table S10. DEGs involved in flower regulation pathways.

Table S11. Nineteen primers used for qPCR analysis.

Table S12. KEGG pathway enrichment of DAMs in flower development in *P. cablin*.

Table S13. Variable importance in projection (VIP) and p-value of four predominant DAMs in the three comparison sets. Student's t test and adjusted p-value (FDR) cutoff: 0.05.

Table S14. Coexpression analysis of flower-related genes.

Table S15. Correlation analysis of the transcriptome and metabolome.

Table S16. The terpenoid contents in *P. cablin* during flower development.

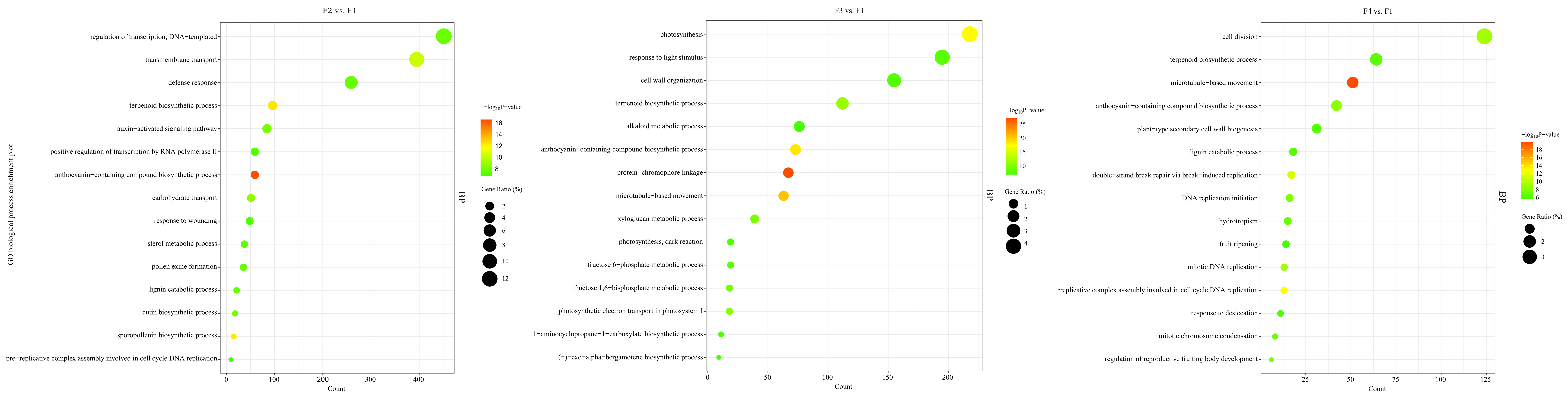
F2 vs. F1
GO biological process enrichment plot
regulation of transcription, DNA−templated
transmembrane transport
defense response
terpenoid biosynthetic process
auxin−activated signaling pathway
positive regulation of transcription by RNA polymerase II
anthocyanin−containing compound biosynthetic process
carbohydrate transport
response to wounding
sterol metabolic process
pollen exine formation
lignin catabolic process
cutin biosynthetic process
sporopollenin biosynthetic process
pre−replicative complex assembly involved in cell cycle DNA replication
0 100 200 300 400
Count
BP
−log10P−value
16 14 12 10 8
Gene Ratio (%)
2 4 6 8 10 12
F3 vs. F1
photosynthesis
response to light stimulus
cell wall organization
terpenoid biosynthetic process
alkaloid metabolic process
anthocyanin−containing compound biosynthetic process
protein−chromophore linkage
microtubule−based movement
xyloglucan metabolic process
photosynthesis, dark reaction
fructose 6−phosphate metabolic process
fructose 1,6−bisphosphate metabolic process
photosynthetic electron transport in photosystem I
1−aminocyclopropane−1−carboxylate biosynthetic process
(−)−exo−alpha−bergamotene biosynthetic process
0 50 100 150 200
Count
BP
−log10P−value
25 20 15 10
Gene Ratio (%)
1 2 3 4
F4 vs. F1
cell division
terpenoid biosynthetic process
microtubule−based movement
anthocyanin−containing compound biosynthetic process
plant−type secondary cell wall biogenesis
lignin catabolic process
double−strand break repair via break−induced replication
DNA replication initiation
hydrotropism
fruit ripening
mitotic DNA replication
-replicative complex assembly involved in cell cycle DNA replication
response to desiccation
mitotic chromosome condensation
regulation of reproductive fruiting body development
25 50 75 100 125
Count
BP
−log10P−value
18 16 14 12 10 8 6
Gene Ratio (%)
1 2 3

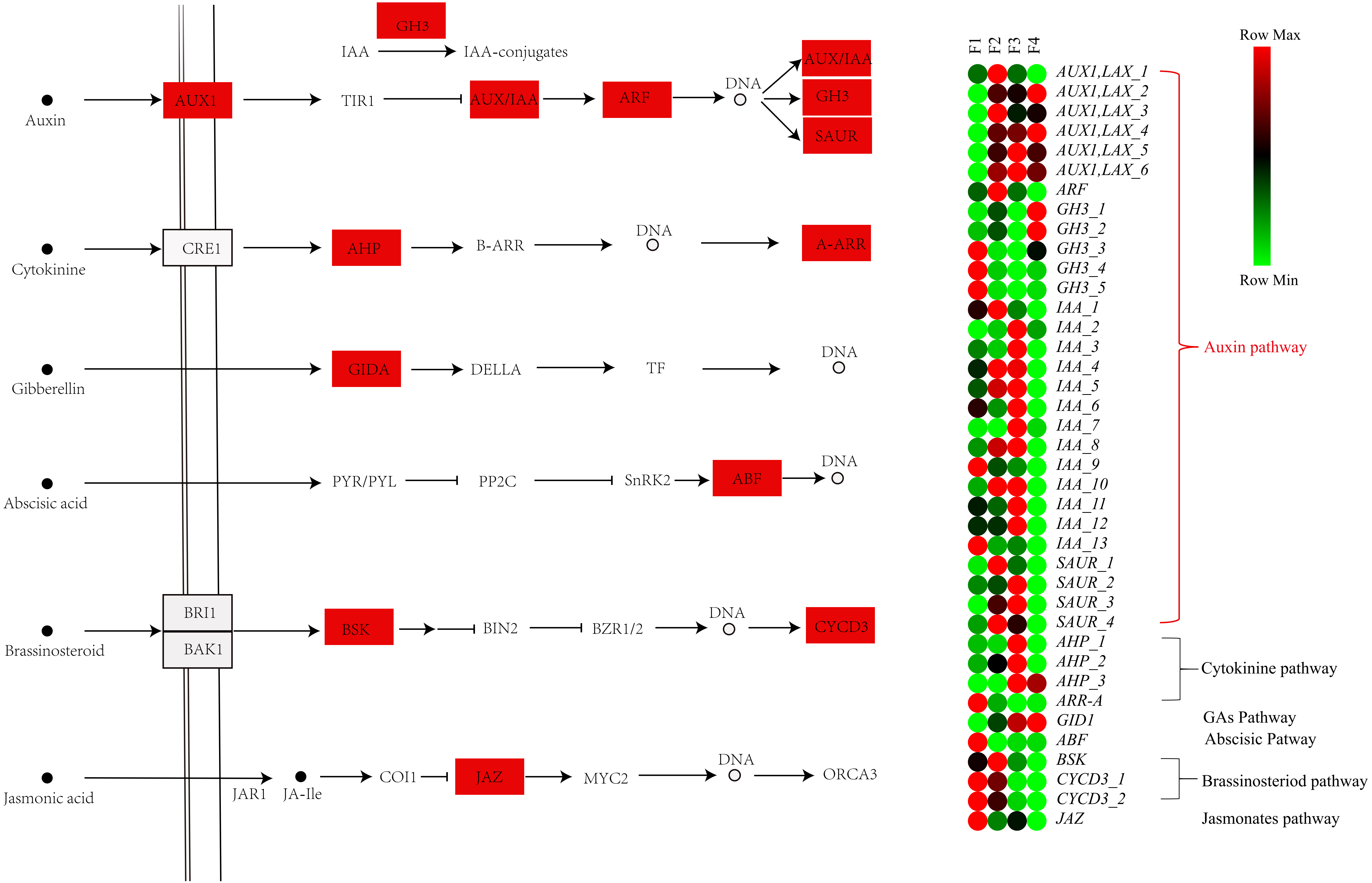

GH3
IAA
IAA-conjugates
Auxin
AUX1
TIR1
AUX/IAA
ARF
DNA
AUX/IAA
GH3
SAUR
Cytokinine
CRE1
AHP
B-ARR
DNA
A-ARR
Gibberellin
GIDA
DELLA
TF
DNA
Abscisic acid
PYR/PYL
PP2C
SnRK2
ABF
DNA
Brassinosteroid
BRI1
BAK1
BSK
BIN2
BZR1/2
DNA
CYCD3
Jasmonic acid
JAR1
JA-Ile
COI1
JAZ
MYC2
DNA
ORCA3
F1
F2
F3
F4
AUX1,LAX_1
AUX1,LAX_2
AUX1,LAX_3
AUX1,LAX_4
AUX1,LAX_5
AUX1,LAX_6
ARF
GH3_1
GH3_2
GH3_3
GH3_4
GH3_5
IAA_1
IAA_2
IAA_3
IAA_4
IAA_5
IAA_6
IAA_7
IAA_8
IAA_9
IAA_10
IAA_11
IAA_12
IAA_13
SAUR_1
SAUR_2
SAUR_3
SAUR_4
AHP_1
AHP_2
AHP_3
ARR-A
GID1
ABF
BSK
CYCD3_1
CYCD3_2
JAZ
Row Max
Row Min
Auxin pathway
Cytokinine pathway
GAs Pathway
Abscisic Patway
Brassinosteriod pathway
Jasmonates pathway

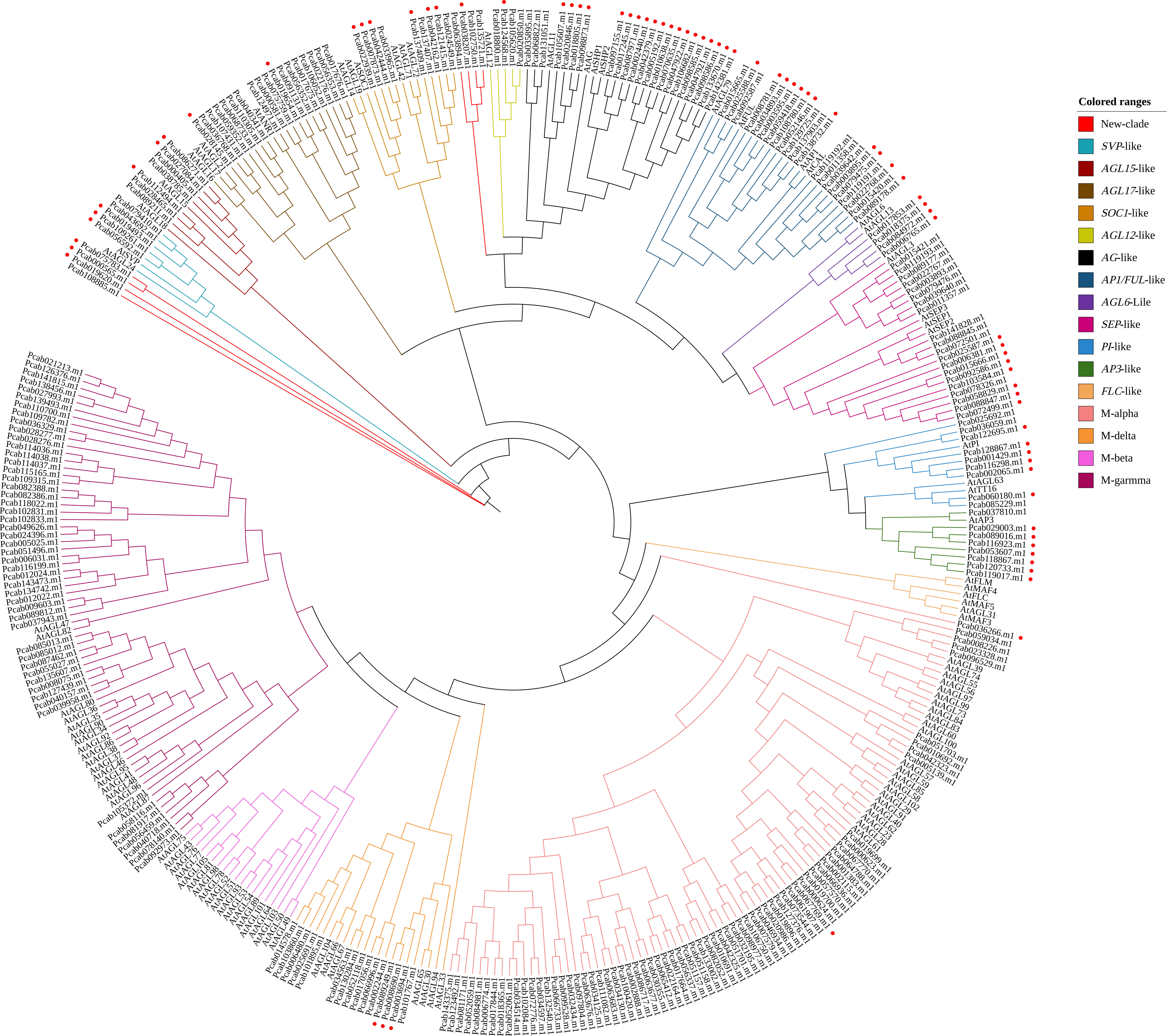
Colored ranges
New-clade
SVP-like
AGL15-like
AGL17-like
SOC1-like
AGL12-like
AG-like
AP1/FUL-like
AGL6-Lile
SEP-like
PI-like
AP3-like
FLC-like
M-alpha
M-delta
M-beta
M-garmma

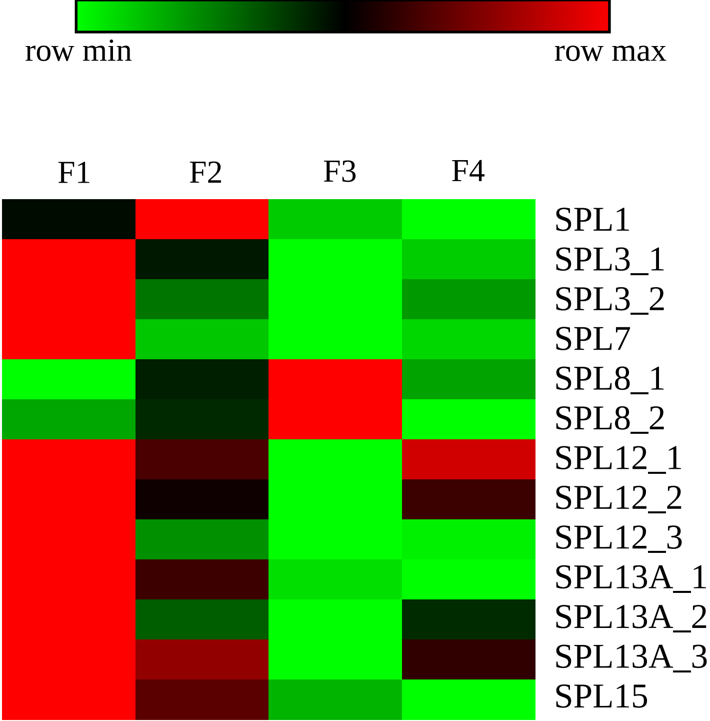
row min
row max
F1
F2
F3
F4
SPL1
SPL3_1
SPL3_2
SPL7
SPL8_1
SPL8_2
SPL12_1
SPL12_2
SPL12_3
SPL13A_1
SPL13A_2
SPL13A_3
SPL15

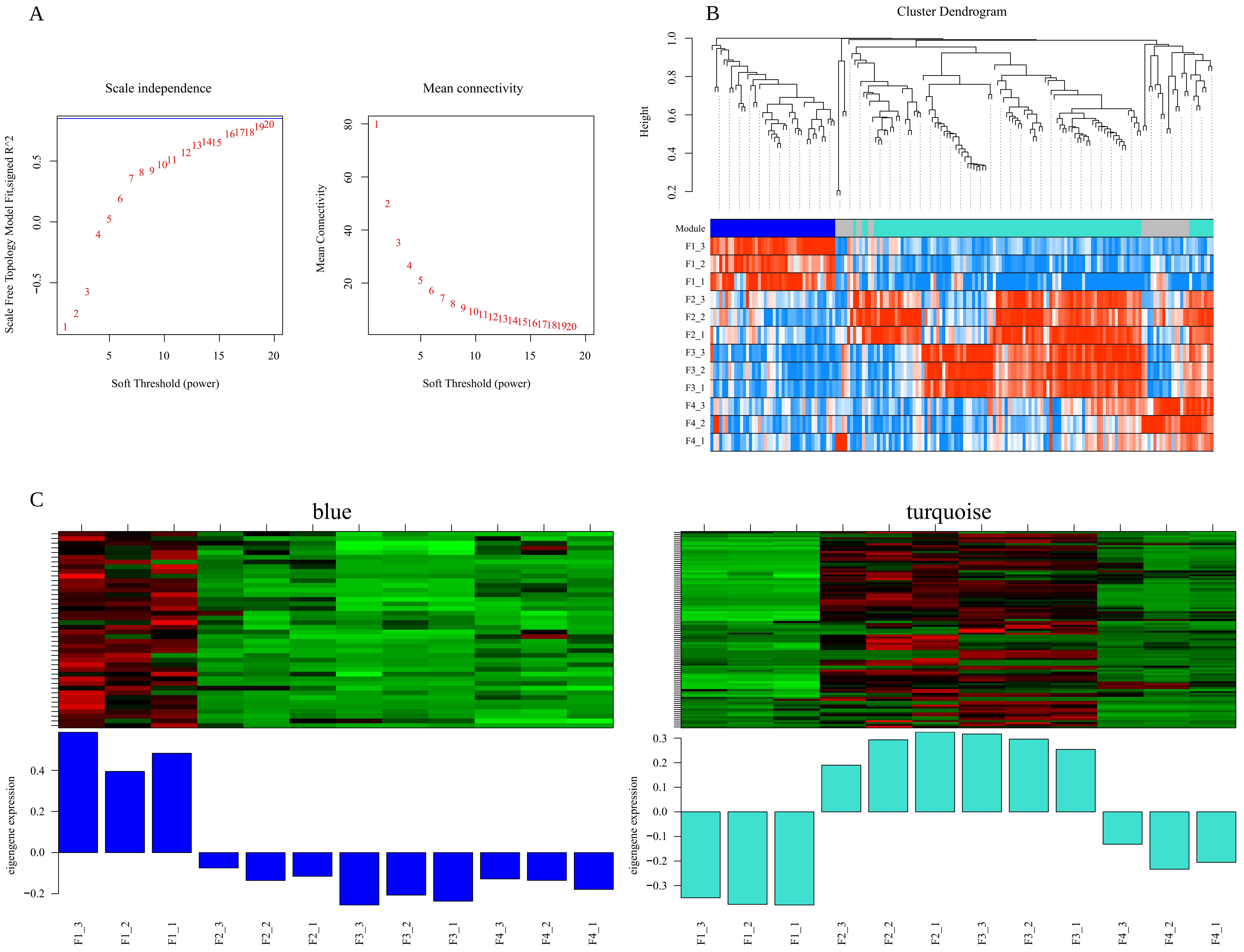

A
Scale independence
Mean connectivity
Scale Free Topology Model Fit,signed R^2
Mean Connectivity
Soft Threshold (power)
Soft Threshold (power)
B
Cluster Dendrogram
Height
Module
F1_3
F1_2
F1_1
F2_3
F2_2
F2_1
F3_3
F3_2
F3_1
F4_3
F4_2
F4_1
C
blue
turquoise
eigengene expression
eigengene expression

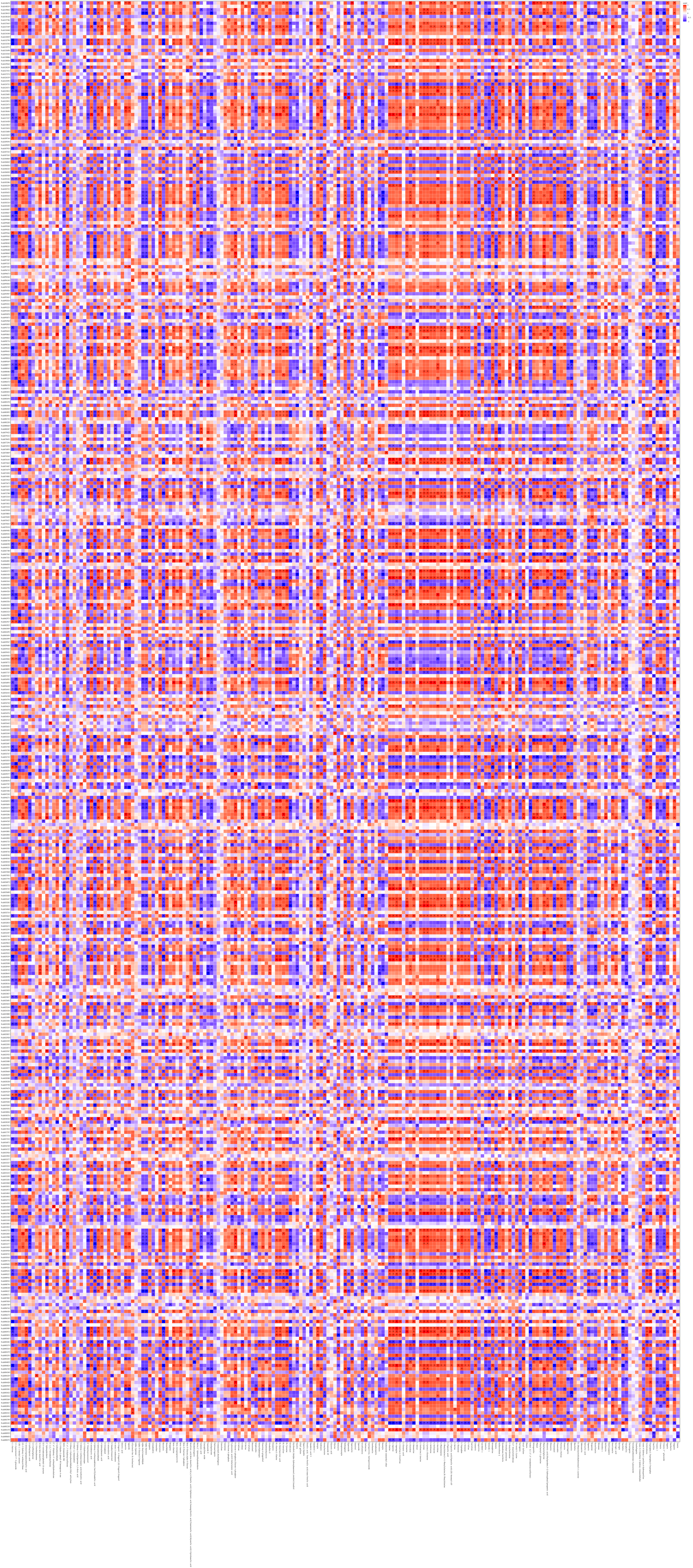

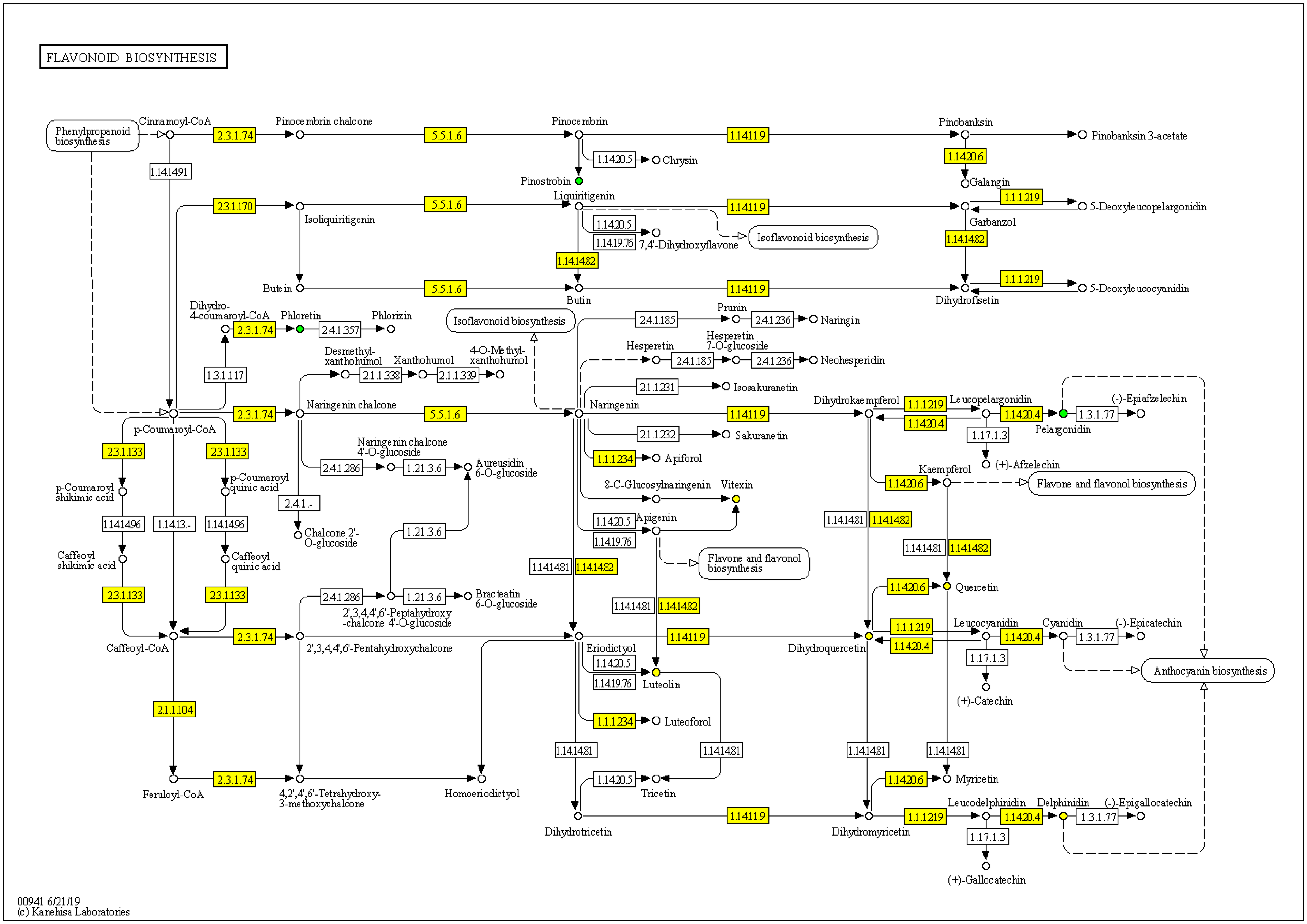
FLAVONOID BIOSYNTHESIS
Phenylpropanoid biosynthesis
Cinnamoyl-CoA
2.3.1.74
Pinocembrin chalcone
5.5.1.6
Pinocembrin
1.14.11.9
Pinobanksin
Pinobanksin 3-acetate
1.14.14.91
1.14.20.5
Chrysin
1.14.20.6
Pinostrobin
Galangin
2.3.1.170
Isoliquiritigenin
Liquiritigenin
1.1.1.219
5-Deoxyleucopelargonidin
Garbanzol
1.14.19.76
7,4'-Dihydroxyflavone
Isoflavonoid biosynthesis
1.14.14.82
Butein
Butin
Dihydrofisetin
5-Deoxyleucocyanidin
Dihydro-4-coumaroyl-CoA
Phloretin
2.4.1.357
Phlorizin
2.4.1.185
Prunin
2.4.1.236
Naringin
Hesperetin
Hesperetin 7-O-glucoside
Neohesperidin
1.3.1.117
Desmethyl-xanthohumol
2.1.1.338
Xanthohumol
2.1.1.339
4-O-Methyl-xanthohumol
2.1.1.231
Isosakuranetin
Naringenin chalcone
Naringenin
Dihydrokaempferol
Leucopelargonidin
(-)-Epiafzelechin
1.14.20.4
1.3.1.77
Pelargonidin
p-Coumaroyl-CoA
2.1.1.232
Sakuranetin
1.17.1.3
2.3.1.133
Naringenin chalcone 4'-O-glucoside
2.4.1.286
1.21.3.6
Aureusidin 6-O-glucoside
1.1.1.234
Apiforol
(+)-Afzelechin
Kaempferol
p-Coumaroyl shikimic acid
p-Coumaroyl quinic acid
2.4.1.-
8-C-Glucosylnaringenin
Vitexin
Flavone and flavonol biosynthesis
1.14.14.96
1.14.13.-
Chalcone 2'-O-glucoside
Apigenin
1.14.14.81
Caffeoyl shikimic acid
Caffeoyl quinic acid
Quercetin
2',3,4,4',6'-Peptahydroxy-chalcone 4'-O-glucoside
Bracteatin 6-O-glucoside
Caffeoyl-CoA
2',3,4,4',6'-Pentahydroxychalcone
Eriodictyol
Dihydroquercetin
Leucocyanidin
Cyanidin
(-)-Epicatechin
Luteolin
Anthocyanin biosynthesis
(+)-Catechin
2.1.1.104
Luteoforol
Myricetin
Feruloyl-CoA
4,2',4',6'-Tetrahydroxy-3-methoxychalcone
Homoeriodictyol
Tricetin
Leucodelphinidin
Delphinidin
(-)-Epigallocatechin
Dihydrotricetin
Dihydromyricetin
(+)-Gallocatechin
00941 6/21/19
(c) Kanehisa Laboratories

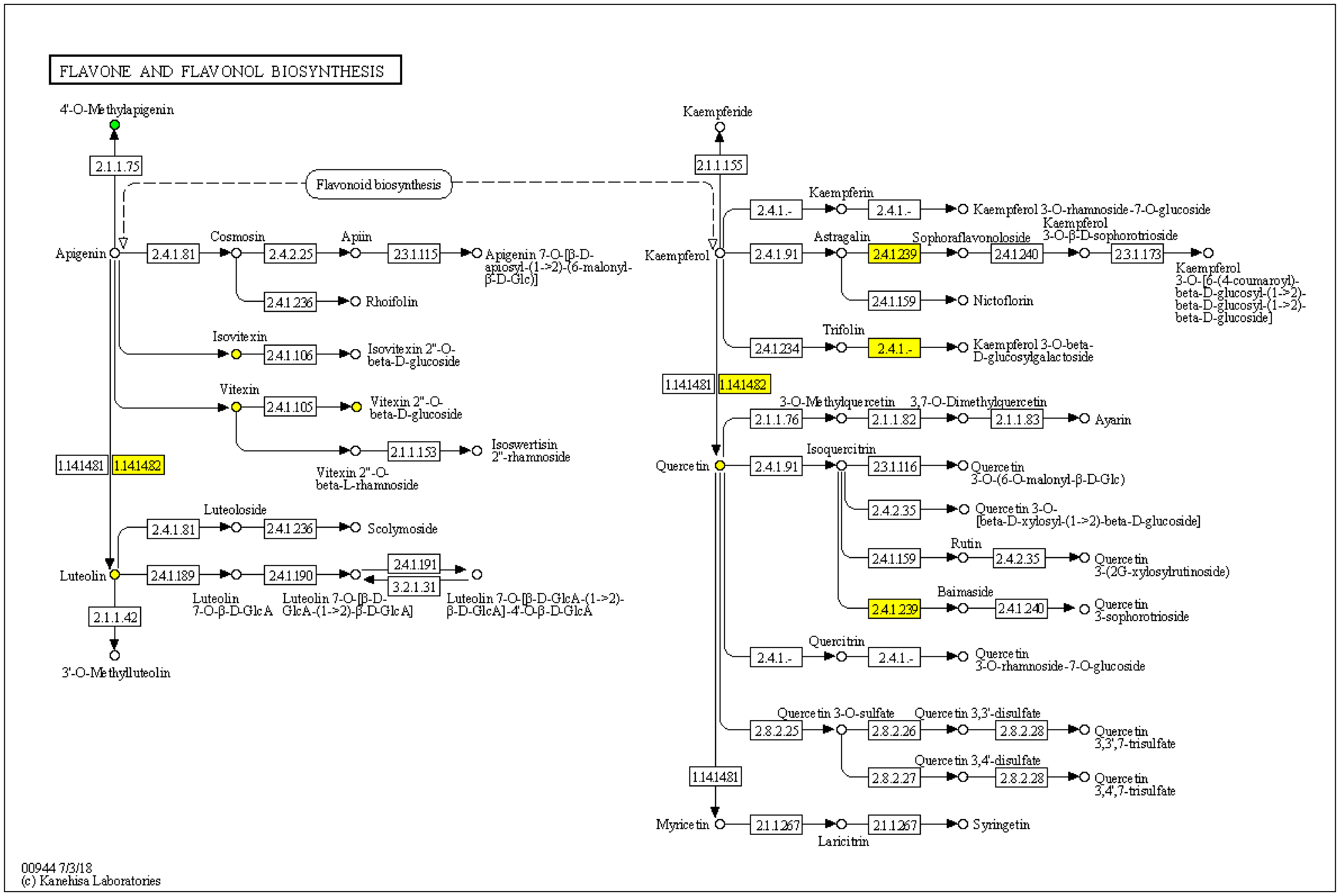
FLAVONE AND FLAVONOL BIOSYNTHESIS
4'-O-Methylapigenin
2.1.1.75
Flavonoid biosynthesis
Apigenin
2.4.1.81
Cosmosin
2.4.2.25
Apiin
2.3.1.115
Apigenin 7-O-[β-D-apiosyl-(1->2)-(6-malonyl-β-D-Glc)]
2.4.1.236
Rhoifolin
Isovitexin
2.4.1.106
Isovitexin 2''-O-beta-D-glucoside
Vitexin
2.4.1.105
Vitexin 2''-O-beta-D-glucoside
2.1.1.153
Isoswertisin 2''-rhamnoside
Vitexin 2''-O-beta-L-rhamnoside
1.14.14.81
1.14.14.82
Luteoloside
2.4.1.81
2.4.1.236
Scolymoside
Luteolin
2.4.1.189
2.4.1.190
2.4.1.191
3.2.1.31
Luteolin 7-O-β-D-GlcA
Luteolin 7-O-[β-D-GlcA-(1->2)-β-D-GlcA]
Luteolin 7-O-[β-D-GlcA-(1->2)-β-D-GlcA]-4'-O-β-D-GlcA
2.1.1.42
3'-O-Methylluteolin
Kaempferide
2.1.1.155
Kaempferin
2.4.1.-
2.4.1.-
Kaempferol 3-O-rhamnoside-7-O-glucoside
Kaempferol
2.4.1.91
Astragalin
2.4.1.239
Sophoraflavonoloside
2.4.1.240
Kaempferol 3-O-β-D-sophorotrioside
2.3.1.173
Kaempferol 3-O-[6-(4-coumaroyl)-beta-D-glucosyl-(1->2)-beta-D-glucosyl-(1->2)-beta-D-glucoside]
2.4.1.159
Nictoflorin
Trifolin
2.4.1.234
2.4.1.-
Kaempferol 3-O-beta-D-glucosylgalactoside
1.14.14.81
1.14.14.82
3-O-Methylquercetin
3,7-O-Dimethylquercetin
2.1.1.76
2.1.1.82
2.1.1.83
Ayarin
Quercetin
2.4.1.91
Isoquercitrin
2.3.1.116
Quercetin 3-O-(6-O-malonyl-β-D-Glc)
2.4.2.35
Quercetin 3-O-[beta-D-xylosyl-(1->2)-beta-D-glucoside]
Rutin
2.4.1.159
2.4.2.35
Quercetin 3-(2G-xylosylrutinoside)
Baimaside
2.4.1.239
2.4.1.240
Quercetin 3-sophorotrioside
Quercitrin
2.4.1.-
2.4.1.-
Quercetin 3-O-rhamnoside-7-O-glucoside
Quercetin 3-O-sulfate
Quercetin 3,3'-disulfate
2.8.2.25
2.8.2.26
2.8.2.28
Quercetin 3,3',7-trisulfate
Quercetin 3,4'-disulfate
2.8.2.27
2.8.2.28
Quercetin 3,4',7-trisulfate
1.14.14.81
Myricetin
2.1.1.267
2.1.1.267
Syringetin
Laricitrin
00944 7/3/18
(c) Kanehisa Laboratories

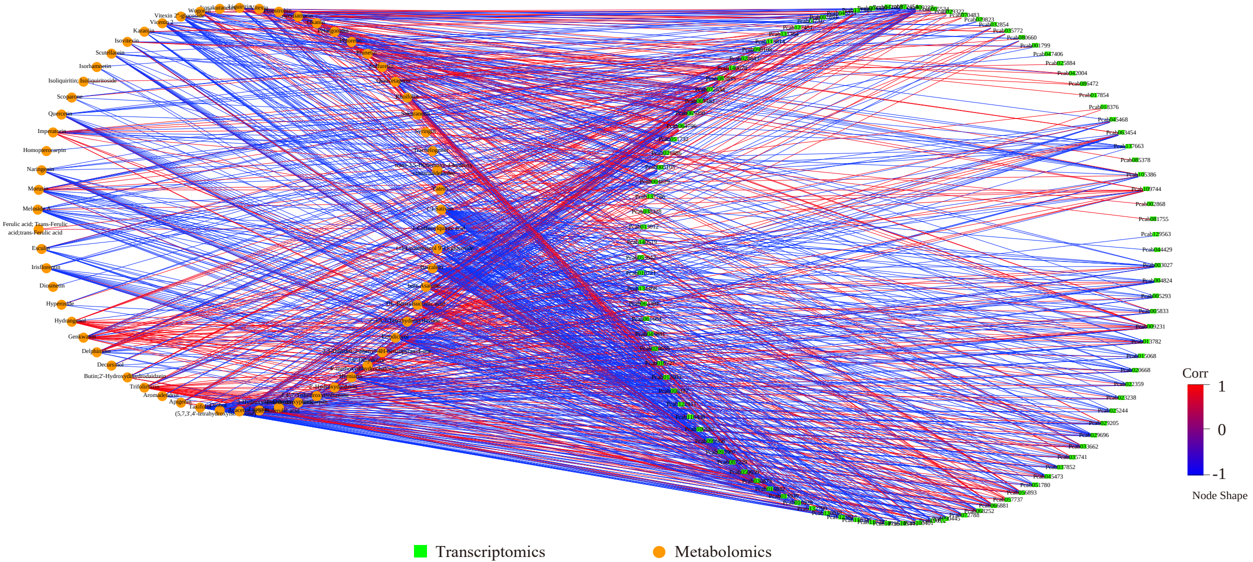

Corr
1
0
-1
Node Shape
Transcriptomics
Metabolomics
Wogonin
Vitexin 2"-glucoside
Vicenin 2
Karanjin
Isovitexin
Scutellarein
Isorhamnetin
Isoliquiritin; Isoliquiritoside
Scoparone
Quercetin
Imperatorin
Homopterocarpin
Naringenin
Morusin
Meloside A
Ferulic acid; Trans-Ferulic acid;trans-Ferulic acid
Esculin
Irisflorentin
Diosmetin
Hyperoside
Hydrangenol
Genkwanin
Delphinidin
Decursinol
Butin;2'-Hydroxydihydrodaidzein
Trifolirhizin
Aromadendrin
Apigenin
Liquiritin
Vitexin
Okanin
Pelargonidin
Quercetagetin
Rhoifolin
Syringin
Baicalein

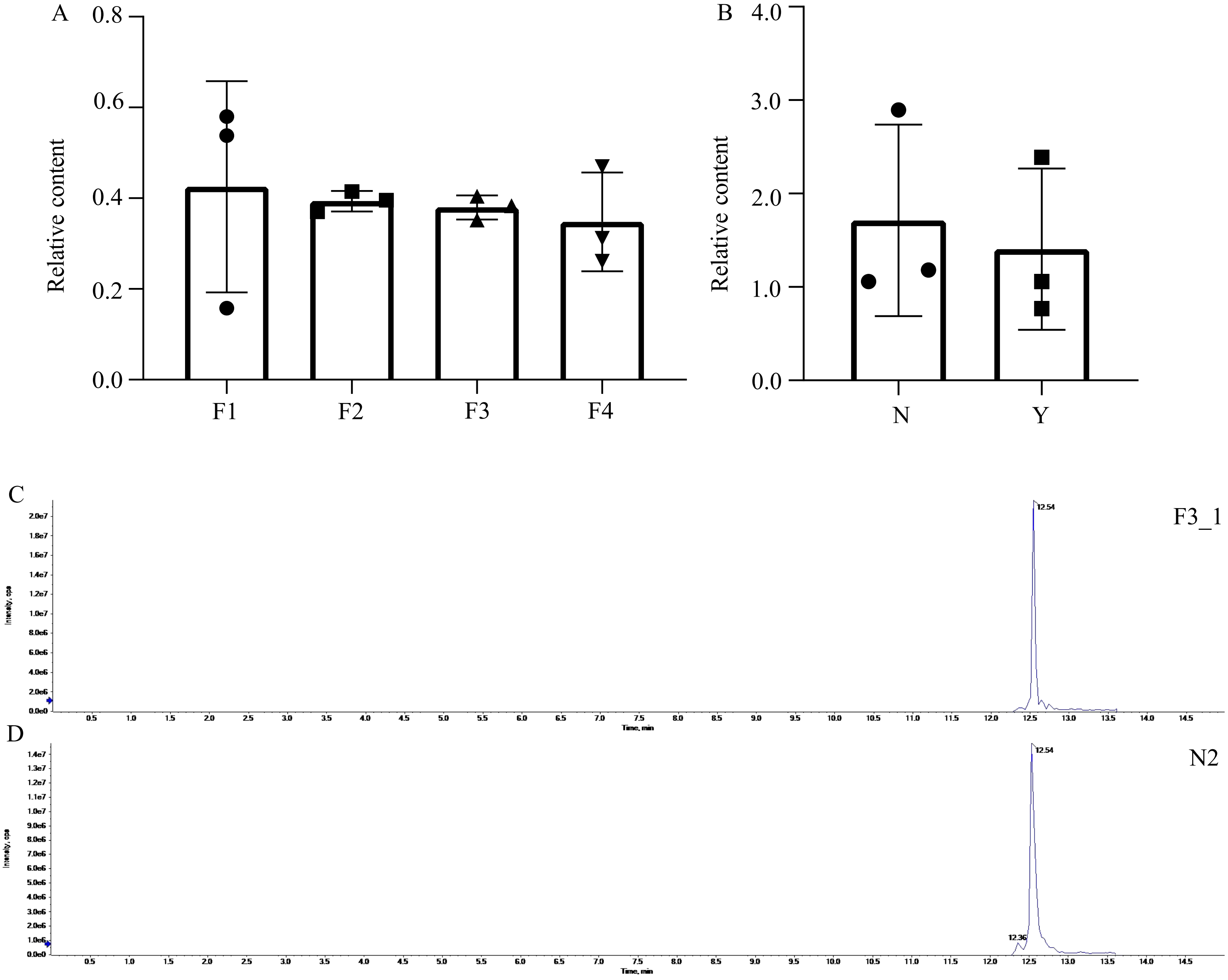
A
B
Relative content
0.0
0.2
0.4
0.6
0.8
F1
F2
F3
F4
1.0
2.0
3.0
4.0
N
Y
C
F3_1
D
N2
12.54
12.36
Intensity, cps
Time, min
